# Two New Families of Local Asymptotically Minimax Lower Bounds in Parameter Estimation

**DOI:** 10.3390/e26110944

**Published:** 2024-11-04

**Authors:** Neri Merhav

**Affiliations:** The Viterbi Faculty of Electrical and Computer Engineering, Technion—Israel Institute of Technology, Technion City, Haifa 3200003, Israel; merhav@ee.technion.ac.il; Tel.: +972-4-8294737

**Keywords:** parameter estimation, minimax estimation, mean-square error, hypothesis testing, probability of error, Fisher information

## Abstract

We propose two families of asymptotically local minimax lower bounds on parameter estimation performance. The first family of bounds applies to any convex, symmetric loss function that depends solely on the difference between the estimate and the true underlying parameter value (i.e., the estimation error), whereas the second is more specifically oriented to the moments of the estimation error. The proposed bounds are relatively easy to calculate numerically (in the sense that their optimization is over relatively few auxiliary parameters), yet they turn out to be tighter (sometimes significantly so) than previously reported bounds that are associated with similar calculation efforts, across many application examples. In addition to their relative simplicity, they also have the following advantages: (i) Essentially no regularity conditions are required regarding the parametric family of distributions. (ii) The bounds are local (in a sense to be specified). (iii) The bounds provide the correct order of decay as functions of the number of observations, at least in all the examples examined. (iv) At least the first family of bounds extends straightforwardly to vector parameters.

## 1. Introduction

The theory of parameter estimation consists of a very large plethora of lower bounds (as well as upper bounds), which characterize the fundamental performance limits of any estimator in a given parametric model. In this context, it is common to distinguish between Bayesian bounds (see, e.g., the Bayesian Cramér–Rao bound [1], the Bayesian Bhattacharyya bound, the Bobrovsky–Zakai bound [2], the Bellini–Tartara bound [3], the Chazan–Zakai–Ziv bound [4], the Weiss–Weinstein bound [5,6], and more (see [7] for a comprehensive overview), and non-Bayesian bounds, where in the former, the parameter to be estimated is considered a random variable with a given probability law, as opposed to the latter, where it is assumed an unknown deterministic constant. The category of non-Bayesian bounds is further subdivided into two subclasses; one is associated with local bounds that hold for classes of estimators with certain limitations, such as unbiased estimators (see, e.g., the Cramér–Rao bound [8,9,10,11,12], the Bhattacharyya bound [13], the Barankin bound [14], the Chapman–Robbins bound [15], the Fraser–Guttman bound [16], the Keifer bound [17], and more), and the other is the subclass of minimax bounds (see, e.g., Ziv and Zakai [18], Hajek [19], Le Cam [20], Assouad [21], Fano [22], Lehmann (Sections 4.2–4.4 in [23]), Nazin [24], Yang and Barron [25], Guntuboyina [26,27], Kim [28], and many more).

In this paper, we focus on the minimax approach, and more concretely, on the local minimax approach. According to the minimax approach, we are given a parametric family of probability density functions (or probability mass functions, in the discrete case), $\left\{p\left(x_{1},\ldots ,x_{n}|\theta \right),\;\left(x_{1},\ldots ,x_{n}\right)\in R^{n},\;\theta \in \Theta \right\}$, where θ is a *d*-dimensional parameter vector, $\Theta \subseteq R^{d}$ is the parameter space, *n* is a positive integer designating the number of observations, and we define a loss function, $l(\theta ,{\hat{\theta }}_{n})$, where ${\hat{\theta }}_{n}$ is an estimator, which is a function of the observations $x_{1},\ldots ,x_{n}$ only. The minimax performance is defined as

(1) $$R_{n}\left(\Theta \right)\overset{▵}{=}\underset{{\hat{\theta }}_{n}\left(\cdot \right)}{\inf}\underset{\theta \in \Theta }{\sup}E_{\theta }\left\{l\left(\theta ,{\hat{\theta }}_{n}\right)\right\},$$

where $E_{\theta }$ denotes expectation w.r.t. p(·|θ). As customary, we consider here loss functions with the property that $l(\theta ,{\hat{\theta }}_{n})$ depends on θ and ${\hat{\theta }}_{n}$ only via their difference, that is, $l\left(\theta ,{\hat{\theta }}_{n}\right)=\rho \left(\theta -{\hat{\theta }}_{n}\right)$, where the function ρ(·) satisfies certain assumptions (see Section 2). The local asymptotic minimax performance at the point θ∈Θ is defined as follows (see also, e.g., [19]). Let $\left\{\zeta_{n}^{*},\;n\geq 1\right\}$ be a positive sequence, tending to infinity, with the property that

(2) $$r\left(\theta \right)\overset{▵}{=}\lim_{\delta ↓0}\underset{n\to \infty }{lim inf}\underset{{\hat{\theta }}_{n}\left(\cdot \right)}{\inf}\underset{\left\{\theta^{\prime }:\;∥\theta^{\prime }-\theta ∥\leq \delta \right\}}{\sup}\zeta_{n}^{*}\cdot E_{\theta^{\prime }}\left\{\rho \left({\hat{\theta }}_{n}-\theta^{\prime }\right)\right\}$$

is a strictly positive finite constant. Then, we say that r(θ) is the local asymptotic minimax performance with respect to (w.r.t.) $\left\{\zeta_{n}^{*}\right\}$ at the point θ∈Θ. Roughly speaking, the significance is that the performance of a good estimator, ${\hat{\theta }}_{n}$, at θ is about $E_{\theta }\left\{\rho \left(\theta -{\hat{\theta }}_{n}\right)\right\}\approx r\left(\theta \right)/\zeta_{n}^{*}$. For example, in the scalar mean square error (MSE) case, where $\rho \left(\varepsilon \right)=\varepsilon^{2}$, and where the observations are Gaussian, i.i.d., with mean θ and known variance $\sigma^{2}$, it is actually shown in Example 2.4, p. 257 in [23] that $r\left(\theta \right)=\sigma^{2}$ w.r.t. $\zeta_{n}^{*}=n$, for all θ∈R, which is attained by the sample mean estimator, ${\hat{\theta }}_{n}=\frac{1}{n}\sum_{i=1}^{n}x_{i}$.

Our focus in this work is on the derivation of some new lower bounds that are as follows: (i) essentially free of regularity conditions on the smoothness of the parametric family {p(·|θ),θ∈Θ}, (ii) relatively simple and easy to calculate, at least numerically, which amounts to the property that the bound contains only a small number of auxiliary parameters to be numerically optimized (typically, no more than two or three parameters), (iii) tighter than earlier reported bounds that are associated with similar calculation efforts as described in (ii), and (iv) lend themselves to extensions that yield even stronger bounds (albeit with more auxiliary parameters to be optimized), as well as extensions to vector parameters. We propose two families of lower bounds on $R_{n}\left(\Theta \right)$, along with their local versions, of bounding r(θ), with the four above-described properties. The first applies to any convex, symmetric loss function ρ(·), whereas the second is more specifically oriented to the moments of the estimation error, $\rho \left(\varepsilon \right)={\left|\varepsilon \right|}^{t}$, where *t* is a positive real, not necessarily an integer, with special attention devoted to the MSE case, t=2. For the sake of simplicity and clarity of the exposition, in the first two main sections of the paper (Section 3 and Section 4), our focus is on the case of a scalar parameter, as most of our examples are associated with the scalar case. In Section 5, we extend some of our findings to the vector case.

To put this work in the perspective of earlier work on minimax estimation, we next briefly review some of the basic approaches in this problem area. Admittedly, considering the vast amount of literature on the subject, our review below is by no means exhaustive. For a more comprehensive review, the reader is referred to Kim [28].

First, observe the simple fact that the minimax performance is lower bounded by the Bayesian performance of the same loss function (see, e.g., [1,2,3,4,5,6,7]) for any prior on the parameter, θ, and so, every lower bound on the Bayesian performance is automatically a valid lower bound also on the minimax performance (see Section 4.2 in [23]). Indeed, in Section 2.3 in [26], it is argued that the vast majority of existing minimax lower bounding techniques are based upon bounding the Bayes risk from below w.r.t. some prior. Many of these Bayesian bounds, however, are subjected to certain restrictions and regularity conditions concerning the smoothness of the prior and the family of densities, {p(·|θ),θ∈Θ}.

Dating back to Ziv and Zakai’s 1969 article [18] on parameter estimation, applied mostly in the context of time-delay estimation, this prior puts all its mass equally on two values, $\theta_{0}$ and $\theta_{1}$, of the parameter θ, and considering an auxiliary hypothesis testing problem of distinguishing between the two hypotheses, $H_{0}:\;\theta =\theta_{0}$ and $H_{1}:\;\theta =\theta_{1}$ with equal priors (see Section 2 for exact definitions). A simple argument regarding the sub-optimality of a decision rule that is based on estimating θ and deciding on the hypothesis with the closer value of θ, combined with Chebychev’s inequality, yields a simple lower bound on the corresponding Bayes risk, and hence also the minimax risk, in terms of the probability of error of the optimal decision rule. Five years later, Bellini and Tartara [3], and then independently, Chazan, Zakai, and Ziv [4], improved the bound of [18] using somewhat different arguments and obtained Bayesian bounds that apply to the uniform prior. These bounds are also given in terms of the error probability pertaining to the optimal maximum a posteriori (MAP) decision rule of binary hypothesis testing with equal priors, but this time, it had an integral form. These bounds were demonstrated to be fairly tight in several application examples, but they are rather difficult to calculate in most cases. Shortly before the Bellini–Tartara and the Chazan–Zakai–Ziv articles were published, Le Cam [20] proposed a minimax lower bound, which is also given in terms of the error probability associated with binary hypothesis testing, or equivalently, the total variation between $p(\cdot |\theta_{0})$ and $p(\cdot |\theta_{1})$, under the postulate that the loss function l(·,·) is a metric. We will refer to Le Cam’s bound in a more detailed manner later, in the context of our first proposed bound.

A decade later, Assouad [21] extended Le Cam’s two-point testing bound to multiple points, where instead of just two test points, $\theta_{0}$ and $\theta_{1}$, as before, there are, more generally, *m* test points, $\theta_{0},\theta_{1},\ldots ,\theta_{m-1}$, and correspondingly, the auxiliary hypothesis testing problem consists of *m* hypotheses, $H_{i}:\;\theta =\theta_{i}$, i=0,1,…,m−1, with certain priors (again, to be defined in Section 2). Based on those test points, Assouad devised the so-called hypercube method. Another related bounding technique that is based on multiple test points, and referred to as Fano’s method, amounts to further bounding from below the error probability of multiple hypotheses using Fano’s inequality (see Section 2.10 in [29]). Considering the large number of auxiliary parameters to be optimized when multiple hypotheses are present, these bounds demand heavy computational efforts. Also, Fano’s inequality is often loose, even though it is adequate enough for the purpose of proving converse-to-coding theorems in information theory [29]. In later years, Le Cam [30] and Yu [31] extended Le Cam’s original approach to apply to testing mixtures of densities. More recently, Yang and Barron [25] related the minimax problem to the metric entropy of the parametric family, $\{p\left(\cdot |\theta_{,}\;\theta \in \Theta \right\}$, and Cai and Zhou [32] combined Le Cam’s and Assouad’s methods by considering a larger number of dimensions. Guntuboyina [26,27] pursued a different direction by deriving minimax lower bounds using *f*-divergences.

The outline of this article is as follows. In Section 2, we define the problem setting, provide a few formal definitions along with background, and establish the notation. In Section 3, we develop the first family of bounds, and in Section 4, we present the second family, both for the scalar parameter case. Finally, in Section 5, we extend some of our findings to the vector case.

## 2. Problem Setting, Background, Definitions, and Notation

As outlined in the last paragraph of the Introduction, Section 3 and Section 4 are on the scalar parameter case, whereas in Section 5, we extend some of the results to the case of a vector parameter with dimension *d*. In order to avoid repetition, we formalize the problem and define the notation here for the more general vector case, with the understanding that for the scalar case, all definitions remain the same, except that they are confined to the special case of d=1.

Consider a family of probability density functions (PDFs), {p(·|θ),θ∈Θ}, where θ is a parameter vector of dimension *d* to be estimated, and $\Theta \subseteq R^{d}$ is the parameter space. We denote by $E_{\theta }\left\{\cdot \right\}$ the expectation operator w.r.t. p(·|θ). Let ***X*** $=(X_{1},\ldots ,X_{n})$ be a random vector of observations, governed by p(·|θ) for some θ∈Θ. The support of p(·|θ) is assumed $X^{n}$, the *n*th Cartesian power of the alphabet X of each component $X_{i}$, i=1,…,n. The alphabet X may be a finite set, a countable set, a finite interval, an infinite interval, or the entire real line. In the first two cases, the PDFs should be understood to be replaced by probability mass functions and integrations over the observation space should be replaced by summations. A realization of ***X*** will be denoted by $x=\left(x_{1},\ldots ,x_{n}\right)\in X^{n}$.

An estimator, ${\hat{\theta }}_{n}$, is given by any function of the observation vector, $g_{n}:X^{n}\to \Theta$, that is, ${\hat{\theta }}_{n}=g_{n}\left[x\right]$. Since ***X*** is random, so is the estimate $g_{n}\left[X\right]$, as well as the estimation error, $\varepsilon_{n}\left[X\right]={\hat{\theta }}_{n}-\theta =g_{n}\left[X\right]-\theta$. We associate with every possible vector value, ϵ, of $\varepsilon_{n}\left[X\right]$ a certain loss (or “cost”, or “price”), ρ(ϵ), where ρ(·) is a non-negative function with the following properties: (i) monotonically non-increasing in each component, $\epsilon_{i}$, of ϵ, wherever $\epsilon_{i}<0$, i=1,2,…,d, (ii) monotonically non-decreasing in each component, $\epsilon_{i}$, of ϵ, wherever $\epsilon_{i}\geq 0$, i=1,2,…,d, and (iii) ρ(0)=0.

Referring to Section 3 and Section 4, which deals with the scalar case (d=1), we further adopt the following assumptions regarding the loss function ρ. In Section 3, we assume that ρ(·) is as follows: (iv) convex, and (v) symmetric, i.e., ρ(−ϵ)=ρ(ϵ) for every ϵ. In Section 4, we assume, more specifically, that $\rho \left(\varepsilon \right)={\left|\varepsilon \right|}^{t}$, where *t* is a positive constant, not necessarily an integer. This is a special case of the class of loss functions considered in Section 3, except when t∈(0,1), in which case, ${\left|\varepsilon \right|}^{t}$ is a concave (rather than a convex) function of ε. In Section 5, for the vector extension of the results of Section 3, the above-mentioned symmetry assumption (v) is extended to become radial symmetry; namely, ρ(ϵ) will be assumed to depend on ϵ only via its Euclidean norm, ∥ϵ∥.

The expected cost of an estimator $g_{n}$ at a point θ∈Θ, is defined as

(3) $$R_{n}\left(\theta ,g_{n}\right)\overset{▵}{=}E_{\theta }\left\{\rho \left(g_{n}\left[X\right]-\theta \right)\right\}.$$

The global minimax performance is defined as

(4) $$R_{n}\left(\Theta \right)\overset{▵}{=}\underset{g_{n}}{\inf}\underset{\theta \in \Theta }{\sup}R_{n}\left(\theta ,g_{n}\right).$$

Another related notion is that of local asymptotic minimax performance, defined in Section 1, and repeated here for the sake of completeness. Let $\left\{\zeta_{n}^{*},\;n\geq 1\right\}$ be a positive sequence, tending to infinity, with the property that r(θ), defined as in (2), is a strictly positive finite constant. Then, we say that r(θ) is the local asymptotic minimax performance w.r.t. $\left\{\zeta_{n}^{*}\right\}$ at θ∈Θ. The sequence $\left\{1/\zeta_{n}^{*}\right\}$ is referred to as the convergence rate of the minimax estimator.

Similarly, as in earlier articles on minimax lower bounds, many of our proposed bounds are given in terms of the error probability pertaining to certain auxiliary hypothesis testing problems that are associated with two or more *test points* in the parameter space, and the choice of those test points is subjected to optimization. We therefore provide a few definitions, notation conventions, and background associated with elementary hypothesis testing. For further details, the reader is referred to any one of many textbooks that cover the topic, for example, Van Trees (see Sections 2.2 and 2.3 in [1]), Helstrom (see Chapter III in [33]), or Whalen (see Chapter 5 in [34]).

For given $\theta_{0}$ and $\theta_{1}$, both in Θ, consider the problem of deciding between two possible hypotheses regarding the probability distribution that governs the given vector of observations, ***X***. Under hypothesis $H_{0}$, ***X*** is governed by $p(\cdot |\theta_{0})$, and under hypothesis $H_{1}$, ***X*** is governed by $p(\cdot |\theta_{1})$. Suppose also that the a priori probability of $H_{0}$ to be the actual underlying hypothesis is $\mathrm{Pr}\{H_{0}\}=q$, where 0≤q≤1 is given, and so the a priori probability of $H_{1}$ is the complementary probability, $\mathrm{Pr}\{H_{1}\}=1-q$. When $q=\frac{1}{2}$, we say that the priors are equal; otherwise, the priors are unequal. A decision rule $\Omega =(\Omega_{0},\Omega_{1})$ is a partition of the observation space, $X^{n}$, into two disjoint decision regions, $\Omega_{0}\subseteq X^{n}$ and its complementary region, $\Omega_{1}=\Omega_{0}^{c}$: Given that $X=x\in \Omega_{0}$, we decide in favor of $H_{0}$; otherwise, we decide in favor of $H_{1}$. The probability of error, associated with the hypotheses $H_{0}$ and $H_{1}$, referring to test points $\theta_{0}$ and $\theta_{1}$, respectively, with priors *q* and 1−q, respectively, when using the decision rule Ω, is defined as

(5) $$P_{e}\left(q,\theta_{0},\theta_{1},\Omega \right)=q\cdot \int_{\Omega_{1}}p\left(x|\theta_{0}\right)dx+\left(1-q\right)\cdot \int_{\Omega_{0}}p\left(x|\theta_{1}\right)dx.$$

A well-known elementary result in decision theory asserts that the optimal decision rule, $\Omega^{*}=\left(\Omega_{0}^{*},\Omega_{1}^{*}\right)$, in the sense of minimizing $P_{e}\left(q,\theta_{0},\theta_{1},\Omega \right)$, is given by

(6) $$\begin{array}{ccc} \Omega_{0}^{*} & = & \left\{x\in X^{n}:\;q\cdot p\left(x|\theta_{0}\right)\geq \left(1-q\right)\cdot p\left(x|\theta_{1}\right)\right\} \end{array}$$

(7) $$\begin{array}{ccc} \Omega_{1}^{*} & = & \{x\in X^{n}:\;q\cdot p\left(x|\theta_{0}\right)<\left(1-q\right)\cdot p\left(x|\theta_{1}\right)\}, \end{array}$$

where it should be pointed out that attributing the case of a tie, $q\cdot \left(x|\theta_{0}\right)=\left(1-q\right)\cdot p\left(x|\theta_{1}\right)$, to $\Omega_{0}^{*}$ is completely arbitrary, and could have been attributed alternatively to $\Omega_{1}^{*}$ without affecting the probability of error. Here and in the sequel, the minimum probability of error, associated with $\Omega^{*}$, namely, $P_{e}\left(q,\theta_{0},\theta_{1},\Omega^{*}\right)$, will be denoted more simply by $P_{e}\left(q,\theta_{0},\theta_{1}\right)$. On substituting $\Omega^{*}$ into the above definition of the probability of error, we obtain

(8) $$\begin{array}{ccc} P_{e}\left(q,\theta_{0},\theta_{1}\right) & = & q\cdot \int_{\left\{x:\;q\cdot p\left(x|\theta_{0}\right)<\left(1-q\right)\cdot p\left(x|\theta_{1}\right)\right\}}p\left(x|\theta_{0}\right)dx+\\ & & \left(1-q\right)\cdot \int_{\left\{x:\;q\cdot p\left(x|\theta_{0}\right)\geq \left(1-q\right)\cdot p\left(x|\theta_{1}\right)\right\}}p\left(x|\theta_{1}\right)dx\\ & = & \int_{\left\{x:\;q\cdot p\left(x|\theta_{0}\right)<\left(1-q\right)\cdot p\left(x|\theta_{1}\right)\right\}}q\cdot p\left(x|\theta_{0}\right)dx+\\ & & \int_{\left\{x:\;q\cdot p\left(x|\theta_{0}\right)\geq \left(1-q\right)\cdot p\left(x|\theta_{1}\right)\right\}}\left(1-q\right)\cdot p\left(x|\theta_{1}\right)dx\\ & = & \int_{\left\{x:\;q\cdot p\left(x|\theta_{0}\right)<\left(1-q\right)\cdot p\left(x|\theta_{1}\right)\right\}}\min\left\{q\cdot p\left(x|\theta_{0}\right),\left(1-q\right)p\left(x|\theta_{1}\right)\right\}dx+\\ & & \int_{\left\{x:\;q\cdot p\left(x|\theta_{0}\right)\geq \left(1-q\right)\cdot p\left(x|\theta_{1}\right)\right\}}\min\left\{q\cdot p\left(x|\theta_{0}\right),\left(1-q\right)\cdot p\left(x|\theta_{1}\right)\right\}dx\\ & = & \int_{X^{n}}\min\left\{q\cdot p\left(x|\theta_{0}\right),\left(1-q\right)\cdot p\left(x|\theta_{1}\right)\right\}dx\\ & = & 1-\int_{X^{n}}\max\left\{q\cdot p\left(x|\theta_{0}\right),\left(1-q\right)\cdot p\left(x|\theta_{1}\right)\right\}dx. \end{array}$$

The expression of the second to the last line will appear in some of our lower bounds in the sequel and will be recognized and interpreted as $P_{e}\left(q,\theta_{0},\theta_{1}\right)$. The expression of the last line is the one that extends to multiple hypothesis testing, as will be detailed below.

The auxiliary problem of binary hypothesis testing extends from two hypotheses to a general number, *m*, of hypotheses, associated with *m* test points, $\theta_{0},\theta_{1},\ldots ,\theta_{m-1}$, in the following manner. Under hypothesis $H_{i}$, the observation vector ***X*** is governed by $p(\cdot |\theta_{i})$ and the a priori probability of $H_{i}$ being the underlying true hypothesis, $\mathrm{Pr}\{H_{i}\}$, is denoted $q_{i}$, i=0,1,…,m−1, where $q_{0},q_{1},\ldots ,q_{m-1}$ are given non-negative numbers summing to unity. Here, a decision rule $\Omega =(\Omega_{0},\Omega_{1},\ldots ,\Omega_{m-1})$ is a partition of $X^{n}$ into *m* disjoint regions such that if $x\in \Omega_{i}$, we decide in favor of $H_{i}$, i=0,1,…,m−1. The probability of error associated with Ω, $\theta_{0},\theta_{1},\ldots ,\theta_{m-1}$ and $q=(q_{0},q_{1},\ldots ,q_{m-1})$ is defined as

(9) $$P_{e}\left(q,\theta_{0},\theta_{1},\ldots ,\theta_{m-1},\Omega \right)=\sum_{i=0}^{m-1}q_{i}\int_{\Omega_{i}^{c}}p\left(x|\theta_{i}\right)dx,$$

where $\Omega_{i}^{c}$ is complementary to $\Omega_{i}$. The optimal MAP decision rule $\Omega^{*}$ selects the hypothesis $H_{i}$ whose index *i* maximizes the product $q_{i}\cdot p\left(x|\theta_{i}\right)$ among all i∈{0,1,…,m−1}, for the given ***x***, where ties are broken arbitrarily. The probability of error, associated with $\Omega^{*}$, denoted $P_{e}\left(q,\theta_{0},\theta_{1},\ldots ,\theta_{m-1}\right)$, is well known to be given by

(10) $$P_{e}\left(q,\theta_{0},\theta_{1},\ldots ,\theta_{m-1}\right)=1-\int_{X^{n}}\max\{q_{0}\cdot p\left(x|\theta_{0},q_{1}\cdot p\left(x|\theta_{1}\right),\ldots ,q_{m-1}\cdot p\left(x|\theta_{m-1}\right)\right\}dx.$$

Note that for m>2, this is different from the expression

$$\int_{X^{n}}\min\{q_{0}\cdot p\left(x|\theta_{0},q_{1}\cdot p\left(x|\theta_{1}\right),\ldots ,q_{m-1}\cdot p\left(x|\theta_{m-1}\right)\right\}dx,$$

as the latter can be interpreted as the probability that the index *i* of the true hypothesis $H_{i}$ minimizes (rather than maximizes) the product $q_{i}p\left(x|\theta_{i}\right)$ over i∈{0,1,…,m−1} for the given ***x***. Imagine an observer that, upon observing a realization ***x*** of the random vector ***X***, creates a list of indices {i} with the *k* largest values of $q_{i}p\left(x|\theta_{i}\right)$ for some k<m, and an error is defined as the event where the correct *i* is not in that list. This is referred to as a list error, which is a term borrowed from the fields of coded communication and information theory. The last expression is the probability of list error for k=m−1. We will encounter this expression later in certain versions of our lower bound. This completes the background needed about hypothesis testing.

As described in Section 1, our objective in this work is to derive relatively simple and easily computable lower bounds to r(θ), which are as tight as possible. While many existing lower bounds in the literature are satisfactory in terms of yielding the correct rate of convergence, $1/\zeta_{n}^{*}$, here we wish to improve the bound on the constant factor, r(θ). Many of our examples involve numerical calculations which include optimization over auxiliary parameters and occasionally also numerical integrations. All these calculations were carried out using MATLAB R2019a (9.6.0.1072779) 64-bit (win64).

## 3. Lower Bounds for Convex Symmetric Loss Functions

As explained earlier, here and in Section 4, we consider a scalar parameter, namely, d=1.

**Theorem** **1.** *Let the assumptions of Section 2 be satisfied for d=1 and let ρ(·) be a symmetric convex loss function. Then,*

(11) $$R_{n}\left(\Theta \right)\geq \underset{\theta_{0},\;\theta_{1}\in \Theta }{\sup}\left\{2\cdot \rho \left(\frac{\theta_{1}-\theta_{0}}{2}\right)\cdot \underset{0\leq q\leq 1}{\sup}P_{e}\left(q,\theta_{0},\theta_{1}\right)\right\},$$

*where $P_{e}\left(q,\theta_{0},\theta_{1}\right)$ is defined as in Equation (8).*

**Proof** **of Theorem 1.** For every $\theta_{0},\theta_{1}\in \Theta$ and q∈[0,1],

(12) $$\begin{array}{ccc} R_{n}\left(\Theta \right) & \geq & qE_{\theta_{0}}\left\{\rho \left(g_{n}\left[X\right]-\theta_{0}\right)\right\}+\left(1-q\right)E_{\theta_{1}}\left\{\rho \left(g_{n}\left[X\right]-\theta_{1}\right)\right\}\\ & \overset{\left(a\right)}{=} & \int_{R^{n}}[q\cdot p\left(x|\theta_{0}\right)\rho \left(g_{n}\left[x\right]-\theta_{0}\right)+\\ & & \left(1-q\right)\cdot p\left(x|\theta_{1}\right)\rho \left(\theta_{1}-g_{n}\left[x\right]\right)]dx\\ & \geq & 2\cdot \int_{R^{n}}\min\left\{q\cdot p\left(x|\theta_{0}\right),\left(1-q\right)\cdot p\left(x|\theta_{1}\right)\right\}\times \\ & & \left[\frac{1}{2}\rho \left(g_{n}\left[x\right]-\theta_{0}\right)+\frac{1}{2}\rho \left(\theta_{1}-g_{n}\left[x\right]\right)\right]dx\\ & \overset{\left(b\right)}{\geq } & 2\cdot \int_{R^{n}}\min\left\{q\cdot p\left(x|\theta_{0}\right),\left(1-q\right)\cdot p\left(x|\theta_{1}\right)\right\}\times \\ & & \rho \left(\frac{g_{n}\left[x\right]-\theta_{0}}{2}+\frac{\theta_{1}-g_{n}\left[x\right]}{2}\right)dx\\ & = & 2\cdot \rho \left(\frac{\theta_{1}-\theta_{0}}{2}\right)\cdot \int_{R^{n}}\min\left\{q\cdot p\left(x|\theta_{0}\right),\left(1-q\right)\cdot p\left(x|\theta_{1}\right)\right\}dx\\ & = & 2\cdot \rho \left(\frac{\theta_{1}-\theta_{0}}{2}\right)\cdot P_{e}\left(q,\theta_{0},\theta_{1}\right), \end{array}$$

where (a) is due to the assumed symmetry of ρ(·) and (b) is by its assumed convexity. Since the inequality,

(13) $$R_{n}\left(\Theta \right)\geq 2\cdot \rho \left(\frac{\theta_{1}-\theta_{0}}{2}\right)\cdot P_{e}\left(q,\theta_{0},\theta_{1}\right)$$

applies to every $\theta_{0},\theta_{1}\in \Theta$ and q∈[0,1], it applies, in particular, also to the supremum over these auxiliary parameters. This completes the proof of Theorem 1. □

Before we proceed, two comments are in order:

1. Note that $P_{e}\left(q,\theta_{0},\theta_{1}\right)$ is a concave function of *q* for fixed $\left(\theta_{0},\theta_{1}\right)$, as it can be presented as the minimum among a family of affine functions of *q*, given by $\min_{\Omega }\left[q\cdot P\left(\Omega |\theta_{0}\right)+\left(1-q\right)\cdot P\left(\Omega^{c}|\theta_{1}\right)\right]$, where Ω runs over all possible subsets of the observation space, $X^{n}$. Another way to see why this is true is by observing that $P_{e}\left(q,\theta_{0},\theta_{1}\right)$ is given in the second to the last line of (8) by an integral, whose integrand, $\min\{q\cdot p\left(x|\theta_{0}\right),\left(1-q\right)\cdot p\left(x|\theta_{1}\right)\}$, is concave in *q*. Clearly, $P_{e}\left(q,\theta_{0},\theta_{1}\right)=0$ whenever q=0 or q=1. Thus, $P_{e}\left(q,\theta_{0},\theta_{1}\right)$ is maximized by some *q* between 0 and 1. If $P_{e}\left(q,\theta_{0},\theta_{1}\right)$ is strictly concave in *q*, then the maximizing *q* is unique.
2. Note that the lower bound (11) is tighter than the lower bound of $\rho \left(\frac{\theta_{1}-\theta_{0}}{2}\right)P_{e}\left(\frac{1}{2},\theta_{0},\theta_{1}\right)$, which was obtained in Equations (6)–(9a) in [18], both because of the factor of 2 and because of the freedom to optimize *q* rather than setting q=1/2. In a further development of [18] the factor of 2 was accomplished too, but at the price of assuming that the density of the estimation error is symmetric about the origin (see discussion after (10) therein), which limits the class of estimators to which the bound applies. The factor of 2 and the degree of freedom *q* are also the two ingredients that make the difference between (11) and the lower bound due to Le Cam [20] (see also [26,28]). In Chapter 2 in [26] Guntuboyina reviews standard bounding techniques, including those of Le Cam, Assouad, and Fano. In particular, in Example 2.3.2 therein, Guntiboyina presents a lower bound in terms of the error probability associated with general priors, given by $\frac{\eta }{2}\cdot P_{e}\left(q,\theta_{0},\theta_{1}\right)$, where in the case of two hypotheses, $\eta =\min_{\vartheta }\left\{\rho \left(\theta_{0}-\vartheta \right)+\rho \left(\theta_{1}-\vartheta \right)\right\}$, in our notation. Now, if ρ is symmetric and monotonically non-decreasing in the absolute error, then the minimizing ϑ is given by $\frac{\theta_{0}+\theta_{1}}{2}$, which yields $\frac{\eta }{2}=\rho \left(\frac{\theta_{1}-\theta_{0}}{2}\right)$ and so, again, the resulting bound is of the same form as (11) except that it lacks the prefactor of 2.

Our first example demonstrates Theorem 1 on a somewhat technical but simple model, with an emphasis on the point that the optimal *q* may differ from 1/2 and that it is therefore useful to maximize w.r.t. *q* in order to improve the bound relative to the choice q=1/2.

**Example** **1.** *Let X be a random variable distributed exponentially according to*

(14) $$p\left(x|\theta \right)=\theta e^{-\theta x},\;\;\;x\geq 0,$$

*and Θ={1,2}, so that the only possibility to select two different values of θ in the lower bound are $\theta_{0}=1$ and $\theta_{1}=2$. In terms of the hypothesis testing problem pertaining to the lower bound, the likelihood ratio test (LRT) is by comparison of $qe^{-x}$ to $\left(1-q\right)\cdot 2e^{-2x}$. Now, if 2(1−q)≤q, or equivalently, $1\geq q\geq \frac{2}{3}$, the decision is always in favor of $H_{0}$, and then $P_{e}\left(q,1,2\right)=1-q$. For $0\leq q<\frac{2}{3}$, the optimal LRT compares X to $x_{0}\left(q\right)=\ln\frac{2(1-q)}{q}$. If $X>x_{0}\left(q\right)$, one decides in favor of $H_{0}$; otherwise, one decides in favor of $H_{1}$. Thus,*

(15) $$\begin{array}{ccc} P_{e}\left(q,1,2\right) & = & q\int_{0}^{x_{0}\left(q\right)}e^{-x}dx+\left(1-q\right)\int_{x_{0}\left(q\right)}^{\infty }2e^{-2x}dx\\ & = & q\left[1-e^{-x_{0}\left(q\right)}\right]+\left(1-q\right)e^{-2x_{0}\left(q\right)}\\ & = & q\left[1-\frac{q}{2(1-q)}\right]+\left(1-q\right){\left[\frac{q}{2(1-q)}\right]}^{2}\\ & = & \frac{q(4-5q)}{4(1-q)}. \end{array}$$

*In summary,*

(16) $$P_{e}\left(q,1,2\right)=\left\{\begin{array}{cc} \frac{q(4-5q)}{4(1-q)} & 0\leq q<\frac{2}{3}\\ 1-q & \frac{2}{3}\leq q\leq 1 \end{array}\right.$$

*It turns out that for $q=\frac{1}{2}$, $P_{e}\left(\frac{1}{2},1,2\right)=\frac{3}{8}=0.375$, whereas the maximum is 0.382, attained at $q=1-\frac{\sqrt{5}}{5}=0.5528$. Thus,*

(17) $$R_{n}\left(\Theta \right)\geq 2\cdot 0.382\cdot \rho \left(\frac{2-1}{2}\right)=0.764\cdot \rho \left(\frac{1}{2}\right).$$

*This concludes Example 1.*

In the above example, we considered just one observation, n=1. From now on, we will refer to the case where n≫1. In particular, the following simple corollary to Theorem 1 yields a local asymptotic minimax lower bound.

**Corollary** **1.** *For a given θ∈Θ and a constant s, let ${\left\{\xi_{n}\right\}}_{n\geq 1}$ denote a positive sequence tending to zero with the property that*

(18) $$\lim_{n\to \infty }\max_{q}P_{e}\left(q,\theta ,\theta +2s\xi_{n}\right)$$

*exists and is given by a strictly positive constant, which will be denoted by $P_{e}^{\infty }\left(\theta ,s\right)$. Also, let*

(19) $$\omega \left(s\right)\overset{▵}{=}\lim_{u\to 0}\frac{\rho (s\cdot u)}{\rho (u)}.$$

*Then, the local asymptotic minimax performance w.r.t. $\zeta_{n}=1/\rho \left(\xi_{n}\right)$ is lower bounded by*

(20) $$r\left(\theta \right)\geq \underset{s\in R}{\sup}\left\{2\omega \left(s\right)\cdot P_{e}^{\infty }\left(\theta ,s\right)\right\}.$$

Corollary 1 is readily obtained from Theorem 1 by substituting $\theta_{0}=\theta$ and $\theta_{1}=\theta +2s\xi_{n}$ in Equation (11), then multiplying both sides of the inequality by $\zeta_{n}=1/\rho \left(\xi_{n}\right)$, and finally, taking the limit inferior of both sides.

Next, we study a few examples of the use of Corollary 1. As in Example 1, we emphasize again in Example 2 below the importance of having the degree of freedom to maximize over the prior *q* rather than to fix $q=\frac{1}{2}$. Also, in all the examples that were examined, the rate of convergence, $1/\zeta_{n}=\rho \left(\xi_{n}\right)$, is the same as the optimal rate of convergence, $1/\zeta_{n}^{*}$. In other words, it is tight in the sense that there exists an estimator (for example, the maximum likelihood estimator) for which $R_{n}\left(\theta ,g_{n}\right)$ tends to zero at the same rate. In some of these examples, we compare our lower bound to r(θ) to those of earlier reported results on the same models.

**Example** **2.** *Let $X_{1},\ldots ,X_{n}$ be independently, identically distributed (i.i.d.) random variables, uniformly distributed in the range [0,θ]. In the corresponding hypothesis testing problem of Theorem 1, the hypotheses are $\theta =\theta_{0}$ and $\theta =\theta_{1}>\theta_{0}$ with priors, q and 1−q. There are two cases: If $q/\theta_{0}^{n}<\left(1-q\right)/\theta_{1}^{n}$, or equivalently, $q<\theta_{0}^{n}/\left(\theta_{0}^{n}+\theta_{1}^{n}\right)$, one decides always in favor of $H_{1}$ and so, the probability of error is q. If, on the other hand, $q/\theta_{0}^{n}>\left(1-q\right)/\theta_{1}^{n}$, namely, $q>\theta_{0}^{n}/\left(\theta_{0}^{n}+\theta_{1}^{n}\right)$, we decide in favor of $H_{1}$ whenever $\max_{i}X_{i}>\theta_{0}$ and then an error occurs only if $H_{1}$ is true, yet $\max_{i}X_{i}<\theta_{0}$, which happens with probability $\left(1-q\right){\left(\frac{\theta_{0}}{\theta_{1}}\right)}^{n}$. Thus,*

(21) $$P_{e}\left(q,\theta_{0},\theta_{1}\right)=\left\{\begin{array}{cc} q & q<\frac{\theta_{0}^{n}}{\theta_{0}^{n}+\theta_{1}^{n}}\\ \left(1-q\right){\left(\frac{\theta_{0}}{\theta_{1}}\right)}^{n} & q\geq \frac{\theta_{0}^{n}}{\theta_{0}^{n}+\theta_{1}^{n}} \end{array}\right.=\min\left\{q,\left(1-q\right){\left(\frac{\theta_{0}}{\theta_{1}}\right)}^{n}\right\},$$

*which is readily seen to be maximized by $q=\frac{\theta_{0}^{n}}{\theta_{0}^{n}+\theta_{1}^{n}}$ and then*

(22) $$\max_{q}P_{e}\left(q,\theta_{0},\theta_{1}\right)=\frac{\theta_{0}^{n}}{\theta_{0}^{n}+\theta_{1}^{n}}.$$

*Now, to apply Corollary 1, we let $\theta_{0}=\theta$ and $\theta_{1}=\theta_{0}\left(1+2\sigma /n\right)$, which amounts to $s=\theta_{0}\sigma =\theta \sigma$ and $\xi_{n}=1/n$. Then,*

(23) $$P_{e}^{\infty }\left(\theta ,s\right)=\lim_{n\to \infty }\frac{1}{1+{\left[1+2s/\left(\theta n\right)\right]}^{n}}=\frac{1}{1+e^{2s/\theta }}.$$

*In the case of the MSE criterion, $\rho \left(\varepsilon \right)=\varepsilon^{2}$, we have $\omega \left(s\right)=s^{2}$, and so,*

(24) $$r\left(\theta \right)\geq \underset{s\geq 0}{\sup}\frac{2s^{2}}{1+e^{2s/\theta }}=\theta^{2}\cdot \underset{u\geq 0}{\sup}\frac{u^{2}}{2(1+e^{u})}\approx 0.2414\theta^{2}$$

*w.r.t. $\zeta_{n}=1/\rho \left(\xi_{n}\right)=1/{\left(1/n\right)}^{2}=n^{2}$. This bound will be further improved upon in Section 4.*

*If instead of maximizing w.r.t. q, we select q=1/2, then*

(25) $$P_{e}\left(\frac{1}{2},\theta ,\theta \left(1+\frac{2\sigma }{n}\right)\right)=\frac{1}{2}\cdot {\left[\frac{\theta }{\theta (1+2\sigma /n)}\right]}^{n}\to \frac{1}{2}\cdot e^{-2\sigma }=\frac{1}{2}\cdot e^{-2s/\theta },$$

*and then the resulting bound would become*

(26) $$r\left(\theta \right)\geq \underset{s\geq 0}{\sup}s^{2}e^{-2s/\theta }=\theta^{2}\cdot \underset{u\geq 0}{\sup}\frac{u^{2}e^{-u}}{4}\approx 0.1353\theta^{2}$$

*w.r.t. $\zeta_{n}=n^{2}$. Therefore, the maximization over q plays an important role here in terms of tightening the lower bound to r(θ).*

*More generally, for $\rho \left(\varepsilon \right)={\left|\varepsilon \right|}^{t}$ (t≥1), $\omega \left(s\right)={\left|s\right|}^{t}$, and we obtain*

(27) $$r\left(\theta \right)\geq \theta^{t}\cdot \underset{u\geq 0}{\sup}\frac{2u^{t}}{1+e^{2u}},$$

*w.r.t. $\zeta_{n}=n^{t}$, where the supremum, which is in fact a maximum, can always be calculated numerically for every given t. For large t, the maximizing u is approximately t/2, which yields*

(28) $$r\left(\theta \right)\geq \frac{{\left(t\theta \right)}^{t}}{2^{t-1}\left(1+e^{t}\right)}.$$

*On the other hand, for q=1/2, we end up with*

(29) $$r\left(\theta \right)\geq \underset{s\geq 0}{\sup}s^{t}e^{-2s/\theta }={\left(\frac{t\theta }{2e}\right)}^{t}.$$

*For large t, the bound of q=1/2 is inferior to the bound with the optimal q, by a factor of about 1/2. This concludes Example 2.*

**Example** **3.** *Let $X_{1},X_{2},\ldots ,X_{n}$ be i.i.d. random variables, uniformly distributed in the interval [θ,θ+1]. For the hypothesis testing problem, let $\theta_{1}$ be chosen between $\theta_{0}$ and $\theta_{0}+1$. Clearly, if $\min_{i}X_{i}<\theta_{1}$, the underlying hypothesis is certainly $H_{1}$. Likewise, if $\max_{i}X_{i}>\theta_{0}+1$, the decision is in favor of $H_{0}$ with certainty. Thus, an error can occur only if all $\left\{X_{i}\right\}$ fall in the interval $\left[\theta_{1},\theta_{0}+1\right]$, an event that occurs with probability ${\left(\theta_{0}+1-\theta_{1}\right)}^{n}$. In this event, the best to do is to select the hypothesis with the larger prior with a probability of error given by min{q,1−q}. Thus,*

(30) $$P_{e}\left(q,\theta_{0},\theta_{1}\right)={\left(\theta_{0}+1-\theta_{1}\right)}^{n}\cdot \min\left\{q,1-q\right\},$$

*and so,*

(31) $$\max_{q}P_{e}\left(q,\theta_{0},\theta_{1}\right)=\frac{1}{2}{\left[1-\left(\theta_{1}-\theta_{0}\right)\right]}^{n},$$

*achieved by q=1/2. Now, let us select $\xi_{n}=1/n$, which yields*

(32) $$\lim_{n\to \infty }\max_{q}P_{e}\left(q,\theta_{0},\theta_{1}\right)=\lim_{n\to \infty }\frac{1}{2}\cdot {\left(1-\frac{2s}{n}\right)}^{n}=\frac{1}{2}\cdot e^{-2s}.$$

*For $\rho \left(\varepsilon \right)={\left|\epsilon \right|}^{t}$, (t≥1), we have*

(33) $$\begin{array}{c} r\left(\theta \right)\geq \underset{s\geq 0}{\sup}2s^{t}\cdot \frac{1}{2}e^{-2s}=\underset{s\geq 0}{\sup}s^{t}e^{-2s}={\left(\frac{t}{2e}\right)}^{t} \end{array}$$

*w.r.t. $\zeta_{n}=1/{\left|1/n\right|}^{t}=n^{t}$. For the case of MSE, t=2, $r\left(\theta \right)\geq e^{-2}\approx 0.1353$. The constant 0.1353 should be compared with $\frac{1-1/\sqrt{2}}{128}\approx 0.0023$ (see Example 4.9 in [35]), which is two orders of magnitude smaller. This concludes Example 3.*

**Example** **4.** Let $X_{i}=\theta +Z_{i}$, where $\left\{Z_{i}\right\}$ are i.i.d., Gaussian random variables with zero mean and variance $\sigma^{2}$. Here, for the corresponding binary hypothesis testing problem, the optimal value of q is always $q^{*}=\frac{1}{2}$. This can be readily seen from the concavity of $P_{e}\left(q,\theta_{0},\theta_{1}\right)$ in q and its symmetry around q=1/2, as $P_{e}\left(q,\theta_{0},\theta_{1}\right)=P_{e}\left(1-q,\theta_{0},\theta_{1}\right)$. Since

(34) $$\begin{array}{ccc} P_{e}\left(\frac{1}{2},\theta_{0},\theta_{1}\right) & = & Pr\left\{\sum_{i=1}^{n}Z_{i}\geq \frac{n(\theta_{1}-\theta_{0})}{2}\right\}\\ & = & Pr\left\{\frac{1}{\sigma \sqrt{n}}\sum_{i=1}^{n}Z_{i}\geq \frac{\sqrt{n}\left(\theta_{1}-\theta_{0}\right)}{2\sigma }\right\}\\ & = & Q\left(\frac{\sqrt{n}\left(\theta_{1}-\theta_{0}\right)}{2\sigma }\right), \end{array}$$

*where*

(35) $$Q\left(t\right)\overset{▵}{=}\int_{t}^{\infty }\frac{e^{-u^{2}/2}du}{\sqrt{2\pi }},$$

*we select $\xi_{n}=\frac{1}{\sqrt{n}}$, which yields $\theta_{1}-\theta_{0}=\frac{2s}{\sqrt{n}}$*

(36) $$P_{e}^{\infty }\left(\theta ,s\right)=Q\left(\frac{s}{\sigma }\right),$$

*and then for the MSE case, $\omega \left(s\right)=s^{2}$,*

(37) $$r\left(\theta \right)\geq \underset{s\geq 0}{\sup}\left\{2s^{2}Q\left(\frac{s}{\sigma }\right)\right\}=\sigma^{2}\cdot \underset{u\geq 0}{\sup}\left\{2u^{2}Q\left(u\right)\right\}\approx 0.3314\sigma^{2}$$

*w.r.t. $\zeta_{n}=1/{\left(1/\sqrt{n}\right)}^{2}=n$, and so, the asymptotic lower bound to $R_{n}\left(\theta ,g_{n}\right)$ is $0.3314\sigma^{2}/n$.*

*We now compare this bound (which will be further improved in Section 4) with a few earlier reported results. In one of the versions of Le Cam’s bound (see Example 4.7 in [35]) for the same model, the lower bound to r(θ) turns out to be $\frac{\sigma^{2}}{24}=0.0417\sigma^{2}$, namely, an order of magnitude smaller. Also, in Example 3.1 in [28], another version of Le Cam’s method yields $r\left(\theta \right)\geq \left(1-\sqrt{1/2}\right)\sigma^{2}/8=0.0366\sigma^{2}$. According to Corollary 4.3 in [36], $r\left(\theta \right)\geq \frac{\sigma^{2}}{8e}=0.046\sigma^{2}$. Yet another comparison is with Theorem 5.9 in [37], where we find an inequality, which in our notation reads as follows:*

(38) $$\underset{\theta \in \Theta }{\sup}P_{\theta }\left\{{\left(g_{n}\left[X\right]-\theta \right)}^{2}\geq \frac{2\alpha \sigma^{2}}{n}\right\}\geq \frac{1}{2}-\alpha \;\;\;\;\alpha \in \left(0,\frac{1}{2}\right).$$

*Combining it with Chebychev’s inequality yields*

(39) $$\underset{\theta \in \Theta }{\sup}E_{\theta }{\left(g_{n}\left[X\right]-\theta \right)}^{2}\geq \frac{\sigma^{2}}{n}\cdot \max_{0\leq \alpha \leq 1/2}\alpha \left(1-2\alpha \right)=\frac{0.125\sigma^{2}}{n}.$$

*In [23] (p. 257), it is shown that when Θ=R, the minimax estimator for this model is the sample mean, and so, in this case, the correct constant in front of $\sigma^{2}$ is actually 1. This concludes Example 4.*

The next example is related to Example 4, as it is based on the use of the central limit theorem (CLT), which means that the Gaussian tail distribution is used here too.

**Example** **5.** *Consider an exponential family,*

(40) $$p\left(x|\theta \right)=\prod_{i=1}^{n}p\left(x_{i}|\theta \right)=\prod_{i=1}^{n}\frac{e^{\theta T(x_{i})}}{Z(\theta )}=\frac{\exp\left\{\theta \sum_{i=1}^{n}T\left(x_{i}\right)\right\}}{Z^{n}\left(\theta \right)}.$$

*where T(·) is a given function and Z(θ) is a normalization function given by*

(41) $$Z\left(\theta \right)=\int_{R}e^{\theta T(x)}dx,$$

*assuming that the integral converges. In the auxiliary binary hypothesis problem, the test statistic is $\sum_{i=1}^{n}T\left(X_{i}\right)$. If q=1/2, $\xi_{n}=1/\sqrt{n}$ and $\theta_{1}-\theta_{0}=2s/\sqrt{n}$, the LRT amounts to examining whether $\sum_{i=1}^{n}\left[T\left(X_{i}\right)-E_{\theta }\left\{T\left(X_{i}\right)\right\}\right]$ is larger than*

$$\frac{s}{\sqrt{n}}\cdot n\frac{d^{2}\ln Z\left(\theta \right)}{d\theta^{2}}=s\sqrt{n}\cdot \frac{d^{2}\ln Z\left(\theta \right)}{d\theta^{2}}.$$

*In this case, the probability of error can be asymptotically assessed using the CLT, which after a simple algebraic manipulation, becomes:*

(42) $$\begin{array}{ccc} P_{e}^{\infty }\left(\theta ,s\right) & = & Q\left(\frac{sd^{2}\ln Z\left(\theta \right)/d\theta^{2}}{\sqrt{d^{2}\ln Z\left(\theta \right)/d\theta^{2}}}\right)\\ & = & Q\left(s\sqrt{\frac{d^{2}\ln Z\left(\theta \right)}{d\theta^{2}}}\right)\\ & = & Q\left(s\sqrt{I(\theta )}\right), \end{array}$$

*where I(θ) is the Fisher information. Thus, for the MSE,*

(43) $$r\left(\theta \right)\geq \underset{s\geq 0}{\sup}2s^{2}Q\left(s\sqrt{I(\theta )}\right)\approx \frac{0.3314}{I(\theta )}$$

*w.r.t. $\zeta_{n}=1/{\left(1/\sqrt{n}\right)}^{2}=n$. This concludes Example 5.*

In several earlier examples, our bound was shown to outperform (sometimes significantly so) earlier reported bounds for the corresponding models. However, to be honest and fair, we should not ignore the fact that there are also situations where our bound may not be tighter than earlier bounds applied to the same model. Such a case is demonstrated in Example 6 below, where our result is compared to those of Ziv and Zakai [18] and Chazan, Zakai, and Ziv [4] in the context of estimating the delay of a known continuous-time signal corrupted by additive white Gaussian noise (for further developments and applications of the Ziv–Zakai and the Chazan–Zakai–Ziv bounds, see, e.g., [38,39,40,41,42,43,44,45] and references therein). Having said that, it should also be kept in mind that our emphasis in this work is on bounds that are relatively simple and easy to calculate (at least numerically), whereas the Chazan–Zakai–Ziv bound, although very tight in many situations, is notoriously difficult to calculate. Indeed, in this setting, the explicit behavior of the resulting complicated bound of [4] is clearly transparent only at high values of the signal-to-noise ratio (SNR)—see Equations (11), (12), and (14) in [4].

**Example** **6.** *Let X(t)=s(t,θ)+N(t), t∈[0,T], where N(t) is additive white Gaussian noise (AWGN) with double-sided spectral density $N_{0}$ and s(t,θ) is a deterministic signal that depends on the unknown parameter, θ. It is assumed that the signal energy, $E=\int_{0}^{T}s^{2}\left(t,\theta \right)dt$, does not depend on θ (which is the case, for example, when θ is a delay parameter of a pulse fully contained in the observation interval, or when θ is the frequency or the phase of a sinusoidal waveform). We further assume that s(t,θ) is at least twice differentiable w.r.t. θ, and that the energies of the first two derivatives are also independent of θ. Then, as shown in Appendix A, for small $|\theta_{1}-\theta_{0}|$,*

(44) $$\begin{array}{ccc} \varrho (\theta_{0},\theta_{1}) & \overset{▵}{=} & \frac{1}{E}\int_{0}^{T}s\left(t,\theta_{0}\right)s\left(t,\theta_{1}\right)dt\\ & = & 1-\frac{{\left(\theta_{1}-\theta_{0}\right)}^{2}}{2E}\int_{0}^{T}{\left[\frac{\partial s(t,\theta )}{\partial \theta }{|}_{\theta =\theta_{0}}\right]}^{2}dt+o(|\theta_{1}-\theta_{0}{|}^{2})\\ & = & 1-\frac{{\left(\theta_{1}-\theta_{0}\right)}^{2}\dot{E}}{2E}+o(|\theta_{1}-\theta_{0}{|}^{2}), \end{array}$$

*where $\dot{E}$ is the energy $\dot{s}\left(t,\theta \right)=\partial s\left(t,\theta \right)/\partial \theta$.*

The optimal LRT in deciding between the two hypotheses is based on the comparison between the correlations, $\int_{0}^{T}X\left(t\right)s\left(t,\theta_{0}\right)dt$ and $\int_{0}^{T}X\left(t\right)s\left(t,\theta_{1}\right)dt$. Again, the optimal value of q is $q^{*}=1/2$. Thus,

(45) $$\begin{array}{ccc} P_{e}\left(\frac{1}{2},\theta_{0},\theta_{1}\right) & = & Q\left(\sqrt{\frac{E}{2N_{0}}\left[1-\varrho (\theta_{0},\theta_{1})\right]}\right)\\ & \approx & Q\left(\sqrt{\frac{E\dot{E}{\left(\theta_{1}-\theta_{0}\right)}^{2}}{4EN_{0}}}\right)\\ & = & Q\left(\sqrt{\frac{\dot{E}}{4N_{0}}}\cdot \left|\theta_{1}-\theta_{0}\right|\right)\\ & = & Q\left(\sqrt{\frac{\dot{P}}{4N_{0}}}\cdot \sqrt{T}\left|\theta_{1}-\theta_{0}\right|\right), \end{array}$$

*where $\dot{P}=\dot{E}/T$ is the power of $\dot{s}\left(t,\theta \right)$. Since we are dealing here with continuous time, instead of a sequence $\xi_{n}$, we use a function, ξ(T), of the observation time, T, which in this case would be $\xi \left(T\right)=\frac{1}{\sqrt{T}}$. Let $\theta_{0}=\theta$ and $\theta_{1}=\theta +\frac{2s}{\sqrt{T}}$. Then,*

(46) $$P_{e}^{\infty }\left(\theta ,s\right)=Q\left(\sqrt{\frac{\dot{P}}{N_{0}}}\cdot \left|s\right|\right),$$

*which, for the MSE case, yields*

(47) $$\begin{array}{ccc} r(\theta ) & \geq & \underset{s\geq 0}{\sup}\left\{2s^{2}Q\left(\sqrt{\frac{\dot{P}}{N_{0}}}\cdot s\right)\right\}\\ & = & \frac{N_{0}}{\dot{P}}\underset{u\geq 0}{\sup}\left\{2u^{2}Q\left(u\right)\right\}\\ & \approx & \frac{0.3314N_{0}}{\dot{P}}. \end{array}$$

*w.r.t. $\zeta \left(T\right)=1/{\left(1/\sqrt{T}\right)}^{2}=T$, which means that the minimax loss is lower bounded by $r\left(\theta \right)/T\geq 0.3314N_{0}/\dot{E}$. This has the same form as the Cramér–Rao lower bound (CRLB), except that the multiplicative factor is 0.3314 rather than 1. In Equation (20) in [18], the bound is of the same form, but with a multiplicative constant of 0.16 for a high signal-to-noise ratio (SNR). However, in [4], the constant of proportionality was improved to 1 in the high SNR limit, just like in the Cramér–Rao lower bound for the same model. The constant 0.3314 will be improved later to 0.4549 (under Example 9 in the sequel), but it will still be below 1.*

*The case where s(t,θ) is not everywhere differentiable w.r.t. θ can be handled in a similar manner, but some caution should be exercised. For example, consider the model,*

(48) X(t)=s(t−θ)+N(t),

*where −∞<t<∞, θ∈[0,T], N(t) is AWGN as before, and s(·) is a rectangular pulse with duration *Δ* and amplitude $\sqrt{E/\Delta }$, E being the signal energy. Here, ϱ(θ,θ+δ)=1−|δ|/Δ; namely, it also includes a linear term in |δ|, not just the quadratic one. This changes the asymptotic behavior of the resulting lower bound to r(θ), which turns out to be $0.7544{\left(N_{0}\Delta /P\right)}^{2}$ w.r.t. $T^{2}$ (namely, a minimax lower bound of $0.7544{\left(N_{0}\Delta /E\right)}^{2}$). It is interesting to compare this bound to the Chapman–Robbins bound for the same model, which is a local bound of the same form but with a multiplicative constant of 0.1602 instead of 0.7544, and which is limited to unbiased estimators. The Chazan–Zakai–Ziv bound [4] for this case is difficult to calculate, but in the high SNR regime, it behaves like $3{\left(N_{0}\Delta /P\right)}^{2}$ w.r.t. $T^{2}$.*

*It is conceivable that the Chazan–Zakai–Ziv bound for estimating the delay of non-differential signals in Gaussian white noise has been improved even further ever since its publication in 1975, but the point of this particular example remains: our bound may not always be the best available bound. Still, since our bound is easy to calculate, it is worth comparing it to other bounds for every given model. This concludes Example 6.*

## 4. Bounds Based on the Minimum Expected Loss over Some Test Points

In this section, we derive our second family of bounds for the scalar case, d=1.

### 4.1. Two Test Points

The following generic, yet conceptually very simple, lower bound assumes neither symmetry nor convexity of the loss function ρ(·). For a given $\left(x,q,\theta_{0},\theta_{1}\right)\in R^{n}\times \left[0,1\right]\times \Theta^{2}$, let us define

(49) $$\psi \left(x,q,\theta_{0},\theta_{1}\right)=\min_{\vartheta }\left\{qp\left(x|\theta_{0}\right)\rho \left(\vartheta -\theta_{0}\right)+\left(1-q\right)p\left(x|\theta_{1}\right)\rho \left(\vartheta -\theta_{1}\right)\right\}.$$

Then,

(50) $$\begin{array}{ccc} R_{n}\left(\Theta \right) & \geq & \underset{\theta_{0},\theta_{1},q}{\sup}qE_{\theta_{0}}\left\{\rho \left(g_{n}\left[X\right]-\theta_{0}\right)\right\}+\left(1-q\right)E_{\theta_{1}}\left\{\rho \left(g_{n}\left[X\right]-\theta_{1}\right)\right\}\\ & = & \underset{\theta_{0},\theta_{1},q}{\sup}\int_{R^{n}}\left[qp\left(x|\theta_{0}\right)\rho \left(g_{n}\left[x\right]-\theta_{0}\right)+\left(1-q\right)p\left(x|\theta_{1}\right)\rho \left(g_{n}\left[x\right]-\theta_{1}\right)\right]dx\\ & \geq & \underset{\theta_{0},\theta_{1},q}{\sup}\int_{R^{n}}\psi \left(x,q,\theta_{0},\theta_{1}\right)dx. \end{array}$$

If we further assume the symmetry of ρ, then it is easy to see that the minimizer $\vartheta^{*}$, which achieves $\psi (x,q,\theta_{0},\theta_{1})$, is always within the interval $\left[\theta_{0},\theta_{1}\right]$. This is because the objective increases monotonically as we move away from the interval $\left[\theta_{0},\theta_{1}\right]$ in either direction. Of course, this simple idea can easily be extended to apply to the weighted sums of more than two points, in principle, but it would become more complicated—see the next subsection for three such points.

If ρ is concave, then the minimizing ϑ is either $\theta_{0}$ or $\theta_{1}$, depending on which of $qp(x|\theta_{0})$ and $\left(1-q\right)p\left(x|\theta_{1}\right)$ is smaller, and the bound becomes

(51) $$R_{n}\left(\Theta \right)\geq \underset{\theta_{0},\theta_{1},q}{\sup}\rho \left(\theta_{1}-\theta_{0}\right)P_{e}\left(q,\theta_{0},\theta_{1}\right).$$

Minimality at the edge points may also happen for some loss functions that are not concave, like the loss function ρ(u)=1{|u|≥Δ}.

The generic lower bound (50) is more general than our first bound in the sense that it does not require convexity or symmetry of ρ, but the downside is that the resulting expressions are harder to deal with directly, as will be seen shortly. For loss functions other than the MSE or general moments of the estimation error, it may not be a trivial task even to derive a closed-form expression of $\psi (x,q,\theta_{0},\theta_{1})$ (i.e., to carry out the minimization associated with the definition of ψ).

For the case of the MSE, $\rho \left(\varepsilon \right)=\varepsilon^{2}$, the calculation of $\psi (x,q,\theta_{0},\theta_{1})$ is straightforward, and it readily yields

(52) $$\psi \left(x,q,\theta_{0},\theta_{1}\right)={\left(\theta_{1}-\theta_{0}\right)}^{2}\cdot \frac{qp\left(x|\theta_{0}\right)\cdot \left(1-q\right)p\left(x|\theta_{1}\right)}{qp\left(x|\theta_{0}\right)+\left(1-q\right)p\left(x|\theta_{1}\right)}.$$

However, it may not be convenient to integrate this function of ***x*** due to the summation at the denominator. One way to alleviate this difficulty is to observe that

(53) $$\begin{array}{ccc} \psi (x,q,\theta_{0},\theta_{1}) & \geq & {\left(\theta_{1}-\theta_{0}\right)}^{2}\cdot \frac{qp\left(x|\theta_{0}\right)\cdot \left(1-q\right)p\left(x|\theta_{1}\right)}{2\max\{qp\left(x|\theta_{0}\right),\left(1-q\right)p\left(x|\theta_{1}\right)\}}\\ & = & \frac{1}{2}\cdot {\left(\theta_{1}-\theta_{0}\right)}^{2}\cdot \min\left\{qp\left(x|\theta_{0}\right),\left(1-q\right)p\left(x|\theta_{1}\right)\right\}, \end{array}$$

which after integration yields again

(54) $$R_{n}\left(\Theta \right)\geq \underset{\theta_{0},\theta_{1},q}{\sup}\left\{\frac{1}{2}{\left(\theta_{1}-\theta_{0}\right)}^{2}\cdot P_{e}\left(q,\theta_{0},\theta_{1}\right)\right\},$$

exactly as in Theorem 1 in the special case of the MSE. This indicates that the bound (50) is at least as tight as the bound of Theorem 1 for the MSE.

It turns out, however, that we can do better than bounding the denominator, $qp\left(x|\theta_{0}\right)+\left(1-q\right)p\left(x|\theta_{1}\right)$, by $2\cdot \max\{qp\left(x|\theta_{0}\right),\left(1-q\right)p\left(x|\theta_{1}\right)\}$ for the purpose of obtaining a more convenient integrand. To this end, we invoke the following lemma, whose proof appears in Appendix B.

**Lemma** **1.** *Let k be a positive integer and let $a_{1},\ldots ,a_{k}$ be positive reals. Then,*

(55) $$\sum_{i=1}^{k}a_{i}=\underset{(r_{1},\ldots ,r_{k})\in S}{\inf}\max\left\{\frac{a_{1}}{r_{1}},\ldots ,\frac{a_{k}}{r_{k}}\right\},$$

*where S is the interior of the k-dimensional simplex, namely, the set of all vectors $\left(r_{1},\ldots ,r_{k}\right)$ with strictly positive components that sum to unity.*

Applying Lemma 1 with the assignments k=2, $a_{1}=qp\left(x|\theta_{0}\right)$, and $a_{2}=\left(1-q\right)p\left(x|\theta_{1}\right)$, we have

(56) $$qp(x|\theta_{0}+\left(1-q\right)p\left(x|\theta_{1}\right)=\underset{r\in (0,1)}{\inf}\max\left\{\frac{qp(x|\theta_{0})}{r},\frac{\left(1-q\right)p\left(x|\theta_{1}\right)}{1-r}\right\}.$$

Thus,

(57) $$\begin{array}{ccc} \psi (x,q,\theta_{0},\theta_{1}) & = & \frac{qp\left(x|\theta_{0}\right)\cdot \left(1-q\right)p\left(x|\theta_{1}\right)}{\inf_{r\in (0,1)}\max\left\{qp\left(x|\theta_{0}\right)/r,\left(1-q\right)p\left(x|\theta_{1}\right)/\left(1-r\right)\right\}}\\ & = & \underset{r\in (0,1)}{\sup}\frac{qp\left(x|\theta_{0}\right)\cdot \left(1-q\right)p\left(x|\theta_{1}\right)}{\max\left\{qp\left(x|\theta_{0}\right)/r,\left(1-q\right)p\left(x|\theta_{1}\right)/\left(1-r\right)\right\}}\\ & = & \underset{r\in (0,1)}{\sup}\left\{\begin{array}{cc} r\left(1-q\right)p\left(x|\theta_{1}\right) & r\leq r^{*}\\ \left(1-r\right)qp\left(x|\theta_{0}\right) & r\geq r^{*} \end{array}\right.\\ & = & \underset{r\in (0,1)}{\sup}\min\left\{r\left(1-q\right)p\left(x|\theta_{1}\right),\left(1-r\right)qp\left(x|\theta_{0}\right)\right\}, \end{array}$$

where

(58) $$r^{*}=\frac{qp(x|\theta_{0})}{qp\left(x|\theta_{0}\right)+\left(1-q\right)p\left(x|\theta_{1}\right)}.$$

Thus, the bound becomes

$$\begin{array}{ccc} R_{n}\left(\Theta \right) & \geq & \underset{\left\{\left(\theta_{0},\theta_{1},q\right)\in \Theta^{2}\times \left(0,1\right)\right\}}{\sup}{\left(\theta_{1}-\theta_{0}\right)}^{2}\times \\ & & \int_{R^{n}}\underset{r\in (0,1)}{\sup}\min\left\{r\left(1-q\right)p\left(x|\theta_{1}\right),\left(1-r\right)qp\left(x|\theta_{0}\right)\right\}dx\\ & \geq & \underset{\left(\theta_{0},\theta_{1},q\right)\in \Theta^{2}\times \left(0,1\right)}{\sup}\underset{r\in (0,1)}{\sup}{\left(\theta_{1}-\theta_{0}\right)}^{2}\times \\ & & \int_{R^{n}}\min\left\{r\left(1-q\right)p\left(x|\theta_{1}\right),\left(1-r\right)qp\left(x|\theta_{0}\right)\right\}dx\\ & = & \underset{\left(\theta_{0},\theta_{1},q,r\right)\in \Theta^{2}\times {\left(0,1\right)}^{2}}{\sup}{\left(\theta_{1}-\theta_{0}\right)}^{2}\cdot \left(q+r-2qr\right)\times \\ & & \int_{R^{n}}\min\left\{\frac{r(1-q)}{q+r-2qr}\cdot p\left(x|\theta_{1}\right),\frac{(1-r)q}{q+r-2qr}\cdot p\left(x|\theta_{0}\right)\right\}dx\\ & = & \underset{\left(\theta_{0},\theta_{1},q,r\right)\in \Theta^{2}\times {\left(0,1\right)}^{2}}{\sup}{\left(\theta_{1}-\theta_{0}\right)}^{2}\cdot \left(q+r-2qr\right)\cdot P_{e}\left(\frac{(1-r)q}{q+r-2qr},\theta_{0},\theta_{1}\right). \end{array}$$

The bound of Theorem 1 for the MSE is obtained as a special case of r=1/2. Therefore, after the optimization over the additional degree of freedom, *r*, the resulting bound cannot be worse than the MSE bound of Theorem 1. In fact, it may strictly improve as we will demonstrate shortly. The choice r=q gives a prior of 1/2 in the error probability factor, and then the maximum of the external factor, q+r−2rq=2q(1−q), is maximized by q=1/2.

**Example** **7.** *To demonstrate the new bound for the MSE, let us revisit Example 2 and see how it improves the multiplicative constant. In that example,*

(59) $$P_{e}\left(\frac{(1-r)q}{q+r-2qr},\theta_{0},\theta_{1}\right)=\min\left\{\frac{(1-r)q}{q+r-2qr},\frac{r(1-q)}{q+r-2qr}\cdot {\left(\frac{\theta_{0}}{\theta_{1}}\right)}^{n}\right\}.$$

*Let us denote $\alpha ={\left(\theta_{0}/\theta_{1}\right)}^{n}$ and recall that α∈(0,1), provided that we select $\theta_{1}>\theta_{0}$. Then,*

(60) $$\left(q+r-2qr\right)\cdot P_{e}\left(\frac{(1-r)q}{q+r-2qr},\theta_{0},\theta_{1}\right)=\min\left\{\left(1-r\right)q,r\left(1-q\right)\alpha \right\}.$$

The maximum w.r.t. q is attained when (1−r)q=r(1−q)α, namely, for $q=q^{*}\overset{▵}{=}\alpha r/\left[1-\left(1-\alpha \right)r\right]$, which yields

(61) $$\max_{q}\left(q+r-2qr\right)\cdot P_{e}\left(\frac{(1-r)q}{q+r-2qr},\theta_{0},\theta_{1}\right)=\frac{\alpha r(1-r)}{1-(1-\alpha )r}.$$

*Let us denote $\beta \overset{▵}{=}1-\alpha \in \left(0,1\right)$. The maximum of r(1−r)/(1−βr) is attained for*

(62) $$r=r^{*}\overset{▵}{=}\frac{1-\sqrt{1-\beta }}{\beta }=\frac{1-\sqrt{\alpha }}{1-\alpha }=\frac{1}{1+\sqrt{\alpha }},$$

*which yields*

(63) $$\begin{array}{ccc} \max_{q,r}\left(q+r-2qr\right)\cdot P_{e}\left(\frac{(1-r)q}{q+r-2qr},\theta_{0},\theta_{1}\right) & = & \underset{r\in (0,1)}{\sup}\frac{\alpha r(1-r)}{1-(1-\alpha )r}\\ & = & \alpha \cdot {\left(\frac{1-\sqrt{\alpha }}{1-\alpha }\right)}^{2}\\ & = & \frac{\alpha }{{\left(1+\sqrt{\alpha }\right)}^{2}}. \end{array}$$

*To obtain a local bound in the spirit of Corollary 1, take $\theta_{0}=\theta$, $\theta_{1}=\theta \left(1+\frac{s}{n\theta }\right)$, which yields $\alpha =e^{-s/\theta }$ in the limit of large n, and so,*

(64) $$r\left(\theta \right)\geq \underset{s\geq 0}{\sup}\frac{s^{2}e^{-s/\theta }}{{\left(1+e^{-s/[2\theta ]}\right)}^{2}}=\theta^{2}\cdot \underset{u\geq 0}{\sup}u^{2}\cdot \frac{e^{-u}}{{\left(1+e^{-u/2}\right)}^{2}}\approx 0.3102\theta^{2},$$

*w.r.t. $\zeta_{n}=n^{2}$, which improves on our earlier bound in Example 2, $r\left(\theta \right)\geq 0.2414\theta^{2}$. This concludes Example 7.*

More generally, for general moments of the estimation error, a similar derivation yields the following:

**Theorem** **2.** *For $\rho \left(\varepsilon \right)={\left|\varepsilon \right|}^{t}$, t≥1 (not necessarily an integer),*

(65) $$R_{n}\left(\Theta \right)\geq \underset{\theta_{0},\theta_{1},q,r}{\sup}{\left|\theta_{1}-\theta_{0}\right|}^{t}\left[{\left(1-r\right)}^{t-1}q+r^{t-1}\left(1-q\right)\right]\cdot P_{e}\left(\frac{{\left(1-r\right)}^{t-1}q}{{\left(1-r\right)}^{t-1}q+r^{t-1}\left(1-q\right)},\theta_{0},\theta_{1}\right).$$

Applying the local version of Theorem 2 to Example 7, we obtain

(66) $$r\left(\theta \right)\geq \theta^{t}\cdot \underset{s>0}{\sup}\frac{s^{t}e^{-s}}{{\left(1+e^{-s/t}\right)}^{t}}$$

w.r.t. $\zeta_{n}=n^{t}$. Changing the optimization variable from *s* to σ=s/t, we end up with

(67) $$\begin{array}{ccc} r\left(\theta \right)\geq {\left(\theta t\right)}^{t}\cdot {\left[\underset{\sigma >0}{\sup}\frac{\sigma }{e^{\sigma }+1}\right]}^{t} & \approx & {\left(0.2785t\theta \right)}^{t}. \end{array}$$

The factor of ${\left(0.2785t\right)}^{t}$ should be compared with ${\left(1/2e\right)}^{t}=0.1839^{t}$ of Example 2, pertaining to the choice q=r=1/2. The gap increases exponentially with *t*. For the maximum likelihood estimator pertaining to Example 2, which is $g_{n}\left[X\right]=\max_{i}X_{i}$, it is easy to show that whenever *t* is an integer,

(68) $$E_{\theta }\left\{\right|\hat{\theta }-\theta {|}^{t}\}=\frac{n!t!\theta^{t}}{(n+t)!}=\frac{t!\theta^{t}}{\left(n+1)(n+2)\cdots (n+t\right)},$$

and so, the asymptotic gap is between t! and ${\left(0.2785t\right)}^{t}$. Considering the Stirling approximation, the ratio between the upper bound and the lower bound is about $\sqrt{2\pi t}\cdot {\left(1.3211\right)}^{t}$.

### 4.2. Three Test Points

The idea behind the bounds of Section 4.1 can be conceptually extended to be based on more than two test points, but the resulting expressions become cumbersome very quickly as the number of test points grows. For three points, however, this is still manageable and can provide improved bounds. Let us select the three test points to be $\theta_{0}-\Delta$, $\theta_{0}$, and $\theta_{0}+\Delta$ for some $\theta_{0}$ and Δ, and let us assign weights *q*, *r*, and w=1−q−r. Consider the bound

(69) $$\begin{array}{ccc} R_{n}\left(\Theta \right) & \geq & qE_{\theta_{0}-\Delta }\left\{\rho \left(g_{n}\left[X\right]-\theta_{0}+\Delta \right)\right\}+rE_{\theta_{0}}\left\{\rho \left(g_{n}\left[X\right]-\theta_{0}\right)\right\}+\\ & & wE_{\theta_{0}+\Delta }\left\{\rho \left(g_{n}\left[X\right]-\theta_{0}-\Delta \right)\right\}\\ & = & \int_{R^{n}}[q\cdot p\left(x|\theta_{0}-\Delta \right)\rho \left(g_{n}\left[x\right]-\theta_{0}+\Delta \right)+r\cdot p\left(x|\theta_{0}\right)\rho \left(g_{n}\left[x\right]-\theta_{0}\right)+\\ & & w\cdot p\left(x|\theta_{0}+\Delta \right)\rho \left(g_{n}\left[x\right]-\theta_{0}-\Delta \right)]dx\\ & \geq & \int_{R^{n}}\Psi \left(x,\theta_{0},\Delta ,q,r\right)dx, \end{array}$$

where

(70) $$\begin{array}{ccc} \Psi (x,\theta_{0},\Delta ,q,r) & = & \min_{\vartheta }\{q\cdot p\left(x|\theta_{0}-\Delta \right)\rho \left(\vartheta -\theta_{0}+\Delta \right)+r\cdot p\left(x|\theta_{0}\right)\rho \left(\vartheta -\theta_{0}\right)+\\ & & w\cdot p\left(x|\theta_{0}+\Delta \right)\rho \left(\vartheta -\theta_{0}-\Delta \right)\}. \end{array}$$

Considering the case of the MSE,

(71) $$\begin{array}{ccc} \Psi (x,\theta_{0},\Delta ,q,r) & = & \min_{\vartheta }\{q\cdot p\left(x|\theta_{0}-\Delta \right){\left(\vartheta -\theta_{0}+\Delta \right)}^{2}+r\cdot p\left(x|\theta_{0}\right){\left(\vartheta -\theta_{0}\right)}^{2}+\\ & & w\cdot p\left(x|\theta_{0}+\Delta \right){\left(\vartheta -\theta_{0}-\Delta \right)}^{2}\} \end{array}$$

can be found in closed-form. Denoting temporarily $a=qp(x|\theta_{0}-\Delta )$, $b=rp(x|\theta_{0})$, and $c=wp(x|\theta_{0}+\Delta )$, $\Psi (x,\theta_{0},\Delta ,q,r)$ is attained by

(72) $$\vartheta =\vartheta^{*}=\frac{a\left(\theta_{0}-\Delta \right)+b\theta_{0}+c\left(\theta_{0}+\Delta \right)}{a+b+c}=\theta_{0}+\frac{(c-a)\Delta }{a+b+c}.$$

On substituting $\vartheta^{*}$ into the sum of squares, we end up with

$$\begin{array}{ccc} \Psi (x,\theta_{0},\Delta ,q,r) & = & \frac{\left[a{\left(b+2c\right)}^{2}+b{\left(c-a\right)}^{2}+c{\left(2a+b\right)}^{2}\right]\Delta^{2}}{{\left(a+b+c\right)}^{2}}\\ & = & \frac{\left(ab+bc+4ac\right)\Delta^{2}}{a+b+c}\\ & = & \frac{\left[qrp\left(x|\theta_{0}-\Delta \right)p\left(x|\theta_{0}\right)+rwp\left(x|\theta_{0}\right)p\left(x|\theta_{0}+\Delta \right)+4qwp\left(x|\theta_{0}-\Delta \right)p\left(x|\theta_{0}+\Delta \right)\right]\Delta^{2}}{qp\left(x|\theta_{0}-\Delta \right)+rp\left(x|\theta_{0}\right)+wp\left(x|\theta_{0}+\Delta \right)}. \end{array}$$

The lower bound on the MSE is then the sum of three integrals

(73) $$\begin{array}{ccc} I_{1} & = & qr\Delta^{2}\cdot \int_{R^{n}}\frac{p\left(x|\theta_{0}-\Delta \right)p\left(x|\theta_{0}\right)dx}{qp\left(x|\theta_{0}-\Delta \right)+rp\left(x|\theta_{0}\right)+wp\left(x|\theta_{0}+\Delta \right)} \end{array}$$

(74) $$\begin{array}{ccc} I_{2} & = & rw\Delta^{2}\cdot \int_{R^{n}}\frac{p\left(x|\theta_{0}\right)p\left(x|\theta_{0}+\Delta \right)dx}{qp\left(x|\theta_{0}-\Delta \right)+rp\left(x|\theta_{0}\right)+wp\left(x|\theta_{0}+\Delta \right)} \end{array}$$

(75) $$\begin{array}{ccc} I_{3} & = & 4qw\Delta^{2}\cdot \int_{R^{n}}\frac{p\left(x|\theta_{0}-\Delta \right)p\left(x|\theta_{0}+\Delta \right)dx}{qp\left(x|\theta_{0}-\Delta \right)+rp\left(x|\theta_{0}\right)+wp\left(x|\theta_{0}+\Delta \right)}. \end{array}$$

**Example** **8.** *Revisiting again Example 2, for a given t>0, let us denote $C\left(t\right)={\left[0,t\right]}^{n}$, and then $p\left(x|\theta_{0}+i\Delta \right)={\left[\theta_{0}+i\Delta \right]}^{-n}\cdot I\{x\in C\left(\theta_{0}+i\Delta \right)$, i=−1,0,1. Then,*

(76) $$\begin{array}{ccc} I_{1} & = & qr\Delta^{2}\cdot \int_{C(\theta_{0}-\Delta )}\frac{{\left(\theta_{0}-\Delta \right)}^{-n}\theta_{0}^{-n}dx}{q{\left(\theta_{0}-\Delta \right)}^{-n}+r\theta_{0}^{-n}+w{\left(\theta_{0}+\Delta \right)}^{-n}}\\ & = & qr\Delta^{2}\cdot \frac{{\left(\theta_{0}-\Delta \right)}^{n}{\left(\theta_{0}-\Delta \right)}^{-n}\theta_{0}^{-n}}{q{\left(\theta_{0}-\Delta \right)}^{-n}+r\theta_{0}^{-n}+w{\left(\theta_{0}+\Delta \right)}^{-n}}\\ & = & \frac{qr\Delta^{2}\theta_{0}^{-n}}{q{\left(\theta_{0}-\Delta \right)}^{-n}+r\theta_{0}^{-n}+w{\left(\theta_{0}+\Delta \right)}^{-n}}\\ & = & \frac{qr\Delta^{2}}{q{\left[\theta_{0}/\left(\theta_{0}-\Delta \right)\right]}^{n}+r+w{\left[\theta_{0}/\left(\theta_{0}+\Delta \right)\right]}^{n}}. \end{array}$$

*For $\Delta =\theta_{0}s/n$, we then have, for large n:*

(77) $$I_{1}∼\frac{\theta_{0}^{2}}{n^{2}}\cdot \frac{qrs^{2}}{qe^{s}+r+we^{-s}}=\frac{\theta_{0}^{2}}{n^{2}}\cdot \frac{qrs^{2}e^{s}}{qe^{2s}+re^{s}+w}.$$

(78) $$\begin{array}{ccc} I_{2} & = & rw\Delta^{2}\int_{C(\theta_{0})}\frac{\theta_{0}^{-n}{\left(\theta_{0}+\Delta \right)}^{-n}dx}{q{\left(\theta_{0}-\Delta \right)}^{-n}I\left\{x\in C\left(\theta_{0}-\Delta \right)\right\}+r\theta_{0}^{-n}+w{\left(\theta_{0}+\Delta \right)}^{-n}}\\ & = & rw\Delta^{2}\cdot \frac{{\left(\theta_{0}-\Delta \right)}^{n}\theta_{0}^{-n}{\left(\theta_{0}+\Delta \right)}^{-n}}{q{\left(\theta_{0}-\Delta \right)}^{-n}+r\theta_{0}^{-n}+w{\left(\theta_{0}+\Delta \right)}^{-n}}+\\ & & rw\Delta^{2}\cdot \frac{\left[\theta_{0}^{n}-{\left(\theta_{0}-\Delta \right)}^{n}\right]\theta_{0}^{-n}{\left(\theta_{0}+\Delta \right)}^{-n}}{r\theta_{0}^{-n}+w{\left(\theta_{0}+\Delta \right)}^{-n}}\\ & = & rw\Delta^{2}\cdot \frac{{\left(1-\Delta /\theta_{0}\right)}^{n}}{q{\left[\left(1+\Delta /\theta_{0}\right)/\left(1-\Delta /\theta_{0}\right)\right]}^{-n}+r{\left(1+\Delta /\theta_{0}\right)}^{n}+w}+\\ & & rw\Delta^{2}\cdot \frac{\left[1-{\left(1-\Delta /\theta_{0}\right)}^{n}\right]}{r{\left(1+\Delta /\theta_{0}\right)}^{n}+w}\\ & ∼ & \frac{\theta_{0}^{2}}{n^{2}}\cdot rws^{2}\left(\frac{e^{-s}}{qe^{2s}+re^{s}+w}+\frac{1-e^{-s}}{re^{s}+w}\right). \end{array}$$

*Similarly,*

(79) $$I_{3}∼\frac{\theta_{0}^{2}}{n^{2}}\cdot \frac{4qws^{2}}{qe^{2s}+re^{s}+w}.$$

*Thus,*

(80) $$\begin{array}{ccc} r(\theta_{0}) & \geq & \theta_{0}^{2}\cdot \underset{s\geq 0}{\sup}\underset{\left(q,r,w\right)\in R_{+}^{3}:\;q+r+w=1}{\sup}s^{2}\cdot \left[\frac{qre^{s}+4qw+rwe^{-s}}{qe^{2s}+re^{s}+w}+\frac{rw(1-e^{-s})}{re^{s}+w}\right]\\ & \approx & 0.4624\theta_{0}^{2}, \end{array}$$

*w.r.t. $\zeta_{n}=n^{2}$, which is a significant improvement of nearly 50% over the previous bound, $0.3102\theta_{0}^{2}$, which was obtained on the basis of two test points, let alone the bound of Theorem 1, which was $0.2414\theta_{0}^{2}$. This concludes Example 8.*

In general, the integrals $I_{1}$, $I_{2}$, and $I_{3}$ are not easy to calculate due to the summations at the denominators of the integrands. One way to proceed is to apply Lemma 1 to the sum of k=3 terms, but this would introduce two additional parameters to be optimized. Returning to the earlier shorthand notation of *a*, *b*, and *c*, a different approach to get rid of summations at the denominators, at the expense of some loss of tightness, is the following:

(81) $$\begin{array}{ccc} \frac{ab+bc+4ac}{a+b+c} & = & \frac{a(b+2c)}{a+b+c}+\frac{c(b+2a)}{a+b+c}\\ & \geq & \frac{a(b+2c)}{a+b+2c}+\frac{c(b+2a)}{2a+b+c}\\ & \geq & \frac{a(b+2c)}{2\max\{a,b+2c\}}+\frac{c(b+2a)}{2\max\{2a+b,c\}}\\ & = & \frac{1}{2}\cdot \left(\min\{a,b+2c\}+\min\{2a+b,c\}\right)\\ & \geq & \frac{1}{2}\cdot \left(\min\{a,b\}+\min\{b,c\}\right)\\ & = & \frac{1}{2}\cdot \left[\min\left\{qp\left(x|\theta_{0}-\Delta \right),rp\left(x|\theta_{0}\right)\right\}+\min\left\{rp\left(x|\theta_{0}\right),wp\left(x|\theta_{0}+\Delta \right)\right\}\right], \end{array}$$

and so,

(82) $$\begin{array}{ccc} R_{n}\left(\Theta \right) & \geq & \frac{\Delta^{2}}{2}[\int_{R^{n}}\min\left\{qp\left(x|\theta_{0}-\Delta \right),rp\left(x|\theta_{0}\right)\right\}dx+\\ & & \int_{R^{n}}\min\left\{rp\left(x|\theta_{0}\right),wp\left(x|\theta_{0}+\Delta \right)\right\}dx]\\ & = & \frac{\Delta^{2}}{2}[\left(q+r\right)P_{e}\left(\frac{q}{q+r},\theta_{0}-\Delta ,\theta_{0}\right)+\\ & & \left(r+w\right)P_{e}\left(\frac{r}{r+w},\theta_{0},\theta_{0}+\Delta \right)]. \end{array}$$

Note that by setting w=0, we recover the bound obtained by integrating (53), and therefore, by optimizing *w*, the resulting bound cannot be worse. A slightly different (better, but more complicated) route in (81) is to apply Lemma 1 with k=2 in the following manner:

(83) $$\begin{array}{ccc} \frac{ab+bc+4ac}{a+b+c} & = & \frac{a(b+2c)}{a+b+c}+\frac{c(b+2a)}{a+b+c}\\ & \geq & \frac{a(b+2c)}{a+b+2c}+\frac{c(b+2a)}{2a+b+c}\\ & = & \frac{a(b+2c)}{\min_{u}\max\left\{a/u,\left(b+2c\right)/\left(1-u\right)\right\}}+\frac{c(b+2a)}{\min_{v}\max\left\{\left(2a+b\right)/\left(1-v\right),c/v\right\}}\\ & = & \max_{u}\min\left\{\left(1-u\right)a,u\left(b+2c\right)\right\}+\max_{v}\min\left\{v\left(2a+b\right),\left(1-v\right)c\right\}\\ & \geq & \max_{u}\min\left\{\left(1-u\right)a,ub\right\}+\max_{v}\min\left\{vb,\left(1-v\right)c\right\}\\ & = & \max_{u}\min\left\{\left(1-u\right)qp\left(x|\theta_{0}-\Delta \right),urp\left(x|\theta_{0}\right)\right\}+\\ & & \max_{v}\min\left\{vrp\left(x|\theta_{0}\right),\left(1-v\right)wp\left(x|\theta_{0}+\Delta \right)\right\}, \end{array}$$

and so,

(84) $$\begin{array}{ccc} R_{n}\left(\Theta \right) & \geq & \Delta^{2}\cdot [\max_{u}\int_{R^{n}}\min\left\{\left(1-u\right)qp\left(x|\theta_{0}-\Delta \right),urp\left(x|\theta_{0}\right)\right\}dx+\\ & & \max_{v}\int_{R^{n}}\min\left\{vrp\left(x|\theta_{0}\right),\left(1-v\right)wp\left(x|\theta_{0}+\Delta \right)\right\}dx]\\ & = & \Delta^{2}\cdot \{\max_{u}\left[\left(1-u\right)q+ur\right]\cdot P_{e}\left(\frac{(1-u)q}{(1-u)q+ur},\theta_{0}-\Delta ,\theta_{0}\right)+\\ & & \max_{v}\left[vr+\left(1-v\right)w\right]\cdot P_{e}\left(\frac{vr}{vr+(1-v)w},\theta_{0},\theta_{0}+\Delta \right)\}. \end{array}$$

We have just proved the following result:

**Theorem** **3.** *For the MSE case,*

(85) $$\begin{array}{ccc} R_{n}\left(\Theta \right) & \geq & \underset{\theta_{0},\Delta ,q,r,w,\left(u,v\right)\in {\left[0,1\right]}^{2}}{\sup}\Delta^{2}\cdot \{\max_{u}\left[\left(1-u\right)q+ur\right]\cdot P_{e}\left(\frac{(1-u)q}{(1-u)q+ur},\theta_{0}-\Delta ,\theta_{0}\right)+\\ & & \max_{v}\left[vr+\left(1-v\right)w\right]\cdot P_{e}\left(\frac{vr}{vr+(1-v)w},\theta_{0},\theta_{0}+\Delta \right)\}. \end{array}$$

*where the maximum over q, r, and w is under the constraints that they are all non-negative and sum to unity.*

**Example** **9.** *Revisiting Example 4, it is natural that both error probabilities would correspond to a prior of 1/2. This dictates the relations,*

(86) $$\begin{array}{c} u=\frac{q}{q+r} \end{array}$$

(87) $$\begin{array}{c} v=\frac{w}{w+r}, \end{array}$$

*and so, the bound becomes*

(88) $$\begin{array}{ccc} R_{n}\left(\Theta \right) & \geq & \max_{\Delta ,q,w,r}\Delta^{2}\left(\frac{2qr}{q+r}+\frac{2wr}{w+r}\right)\cdot Q\left(\frac{\sqrt{n}\Delta }{2\sigma }\right)\\ & = & \left[\max_{s\geq 0}{\left(\frac{2s\sigma }{\sqrt{n}}\right)}^{2}Q\left(s\right)\right]\cdot \max_{\left\{(q,r):\;q\geq 0,\;r\geq 0,\;q+r\leq 1\right\}}\left\{\frac{2qr}{q+r}+\frac{2r(1-q-r)}{1-q}\right\}\\ & = & \frac{\sigma^{2}}{n}\cdot \max_{s\geq 0}\left\{4s^{2}Q\left(s\right)\right\}\cdot 0.6862\\ & = & \frac{\sigma^{2}}{n}\cdot 0.6629\cdot 0.6862\\ & \approx & \frac{0.4549\sigma^{2}}{n}, \end{array}$$

*which is an improvement on the bound of Example 4, which was $0.3314\sigma^{2}/n$. Similarly, in Example 6, for smooth signals, the multiplicative constant also improves from 0.3314 to 0.4549, and for the rectangular pulse, it improves from 0.7544 to 1.0352 (about 37% improvement in all of them). This concludes Example 9.*

**Example** **10.** *Revisiting Example 2, we now have*

(89) $$\begin{array}{ccc} R_{n}\left(\Theta \right) & \geq & \Delta^{2}\cdot \left[\max_{u}\min\left\{\left(1-u\right)q,ur\alpha \right\}+\max_{v}\min\left\{vr,\left(1-v\right)w\alpha \right\}\right]\\ & = & \Delta^{2}\left(\frac{qr\alpha }{q+r\alpha }+\frac{wr\alpha }{r+w\alpha }\right)\\ & = & \Delta^{2}\left(\frac{qr\alpha }{q+r\alpha }+\frac{r(1-q-r)\alpha }{r+(1-q-r)\alpha }\right)\\ & = & \frac{s^{2}}{n^{2}}e^{-s}r\left(\frac{q}{q+re^{-s}}+\frac{1-q-r}{r+\left(1-q-r\right)e^{-s}}\right)\\ & = & \frac{s^{2}}{n^{2}}r\left(\frac{q}{qe^{s}+r}+\frac{1-q-r}{re^{s}+\left(1-q-r\right)}\right), \end{array}$$

*whose maximum is $0.3909/n^{2}$, an improvement relative to the earlier bound of $0.3102/n^{2}$. This concludes Example 10.*

## 5. Extensions to the Vector Case

In this section, we outline extensions of some of our findings in Section 3 and Section 4 to the vector case. Let θ be a parameter vector of dimension *d* and $\Theta \subseteq R^{d}$. Let ρ(ε) be a convex loss function that depends on the *d*-dimensional error vector ε only via its norm, ∥ε∥ (that is, ρ has radial symmetry).

First, observe that Theorem 1 extends verbatim to the vector case, as nothing in the proof of Theorem 1 is based on any assumption or property that holds only if θ is a scalar parameter.

Corollary 1 can also be extended by letting the two test points of the auxiliary hypothesis testing problem, $\theta_{0}$ and $\theta_{1}$, be selected such that the distance between them, $∥\theta_{1}-\theta_{0}∥$, would decay at an appropriate rate as a function of *n*, so as to make the probability of error, $\max_{q}P_{e}\left(q,\theta_{0},\theta_{1}\right)$, converge to a positive constant as n→∞, as was achieved in Corollary 1 for the scalar case. But in the vector case considered now, there is an additional degree of freedom, which is the *direction* of the displacement vector, $\theta_{1}-\theta_{0}$. This direction can now be optimized so as to yield the largest (hence the tightest) lower bound. To be specific, in the vector case, Corollary 1 remains the same except that now *s* should be thought of as a *d*-dimensional vector rather than a scalar, ρ(u) in the denominator of (19) should be replaced by ρ(v·u), where $v\in R^{d}$ is an arbitrary fixed non-zero vector (for example, any unit norm vector in the case of MSE), and the supremum in (20) should be taken over $R^{d}$. To demonstrate this point, we now revisit Example 5, but this time, for the vector case.

**Example** **11.** *Consider the case where $p\left(x|\theta \right)=\prod_{i=1}^{n}p\left(x_{i}|\theta \right)$, with each factor in this product PDF being given by a d-dimensional exponential family,*

(90) $$p\left(x|\theta \right)=\frac{\exp\{\theta^{\prime }T\left(x\right)\}}{Z(\theta )}$$

*where $\theta^{\prime }T\left(x\right)$ is the inner product of the d-dimensional parameter vector θ and a d-dimensional vector of statistics, $T\left(x\right)=(T_{1}\left(x\right),T_{2}\left(x\right),\ldots ,T_{d}\left(x\right))$, and*

(91) $$Z\left(\theta \right)=\int_{R}\exp\left\{\theta^{\prime }T\left(x\right)\right\}dx,$$

*provided that the integral converges. In the above notation, both θ and T(x) are understood to be column vectors and $\theta^{\prime }$ denotes transposition of θ to a row vector. Similarly as in Corollary 1, to obtain a local bound at a given θ, we let $\theta_{0}=\theta$ and $\theta_{1}=\theta +\frac{2s}{\sqrt{n}}$, where $s\in R^{d}$. A simple extension of the derivation in Example 5 (using the CLT) yields*

(92) $$P_{e}^{\infty }\left(\theta ,s\right)=Q\left(\sqrt{s^{\prime }I\left(\theta \right)s}\right),$$

*where s is considered a column vector, the superscript prime denotes vector transposition as before, and $I\left(\theta \right)=\nabla^{2}\ln Z\left(\theta \right)$ is the d×d Fisher information matrix of the exponential family. Considering the case of the MSE, $\rho \left(\epsilon \right)={∥\epsilon ∥}^{2}$ with v being any unit norm vector, we have*

(93) $$w\left(s\right)=\lim_{u\to 0}\frac{{∥s\cdot u∥}^{2}}{{∥v\cdot u∥}^{2}}={∥s∥}^{2},$$

*and then the following lower bound is obtained w.r.t. $1/∥v/\sqrt{n}{∥}^{2}=n$:*

(94) $$\begin{array}{ccc} r(\theta ) & \geq & \underset{s\in R^{d}}{\sup}\left\{{2∥s∥}^{2}Q\left(\sqrt{s^{\prime }I\left(\theta \right)s}\right)\right\}\\ & = & \underset{t\geq 0}{\sup}\left\{2t^{2}\cdot \max_{\left\{s:\;∥s{∥}^{2}=t^{2}\right\}}Q\left(\sqrt{s^{\prime }I\left(\theta \right)s}\right)\right\}\\ & = & \underset{t\geq 0}{\sup}\left\{2t^{2}\cdot Q\left(t\sqrt{\lambda_{\min}\left(\theta \right)}\right)\right\}\\ & = & \frac{1}{\lambda_{\min}\left(\theta \right)}\underset{\tau \geq 0}{\sup}\left\{2\tau^{2}\cdot Q\left(\tau \right)\right\}\\ & \approx & \frac{0.3314}{\lambda_{\min}\left(\theta \right)}, \end{array}$$

*where $\lambda_{\min}\left(\theta \right)$ is the smallest eigenvalue of I(θ). In this example, it is apparent that the chosen direction of the vector s is that of the eigenvector corresponding to the smallest eigenvalue of the Fisher information matrix. This completes Example 11.*

In the vector case considered in this section, it is also instructive to extend the scope from two test points, $\theta_{0}$ and $\theta_{1}$, to multiple test points, $\theta_{0},\theta_{1},\ldots ,\theta_{m-1}$, along with the corresponding weights (or priors), $q_{0},q_{1},\ldots ,q_{m-1}$ (see the exposition at the end of Section 2). Interestingly, this will also lead to new bounds even for the case of m=2 test points.

To this end, we also consider a set of *m* unitary transformation matrices, $T_{0},T_{1},T_{2},\ldots ,$$T_{m-1}$, with the properties: (i) θ∈Θ if and only if $T_{i}\theta \in \Theta$ for all i=0,1,…,m−1, and (ii) $T_{0}+T_{1}+\cdots +T_{m-1}=0$. For example, if d=2, take $T_{i}$ to be matrices of rotation by 2πi/m, i=0,1,…,m−1.

**Theorem** **4.** *Let $\theta_{0},\theta_{1},\ldots ,\theta_{m-1}\in \Theta \subseteq R^{d}$, d≥1 and $q_{0},q_{1},\ldots ,q_{m-1}$ be given, and let $T_{0},T_{1},\ldots T_{m-1}$ be unitary transformations that sum to zero, as described in the above paragraph. Then, for a convex loss function ρ(ε) that depends on ε only via ∥ε∥, we have:*

(95) $$R_{n}\left(\Theta \right)\geq m\cdot \rho \left(\frac{1}{m}\sum_{i=0}^{m-1}T_{i}\theta_{i}\right)\cdot \int_{R^{n}}\min\left\{q_{0}\cdot p\left(x|\theta_{0}\right),\ldots ,q_{m-1}p\left(x|\theta_{m-1}\right)\right\}dx.$$

As explained in Section 2, the integral on the right-hand side can be interpreted as the probability of list error with a list size of m−1.

**Proof** **of Theorem 4.** The proof is a direct extension of the proof of Theorem 1:

$$\begin{array}{ccc} \underset{\theta \in \Theta }{\sup}E_{\theta }\left\{\rho \left(g_{n}\left[X\right]-\theta \right)\right\} & \geq & \sum_{i=0}^{m-1}q_{i}E_{\theta_{i}}\left\{\rho \left(g_{n}\left[X\right]-\theta_{i}\right)\right\}\\ & = & \int_{R^{n}}\left[\sum_{i=0}^{m-1}q_{i}\cdot p\left(x|\theta_{i}\right)\rho \left(g_{n}\left[x\right]-\theta_{i}\right)\right]dx\\ & \geq & m\cdot \int_{R^{n}}\min\left\{q_{0}\cdot p\left(x|\theta_{0}\right),\ldots ,q_{m-1}p\left(x|\theta_{m-1}\right)\right\}\times \\ & & \left[\frac{1}{m}\sum_{i=0}^{m-1}\rho \left(\theta_{i}-g_{n}\left[X\right]\right)\right]dx\\ & \overset{\left(a\right)}{=} & m\cdot \int_{R^{n}}\min\left\{q_{0}\cdot p\left(x|\theta_{0}\right),\ldots ,q_{m-1}p\left(x|\theta_{m-1}\right)\right\}\times \\ & & \left[\frac{1}{m}\sum_{i=0}^{m-1}\rho \left(T_{i}\left(\theta_{i}-g_{n}\left[X\right]\right)\right)\right]dx\\ & \overset{\left(b\right)}{\geq } & m\cdot \int_{R^{n}}\min\left\{q_{0}\cdot p\left(x|\theta_{0}\right),\ldots ,q_{m-1}p\left(x|\theta_{m-1}\right)\right\}\times \\ & & \rho \left(\frac{1}{m}\sum_{i=0}^{m-1}T_{i}\left(\theta_{i}-g_{n}\left[X\right]\right)\right)dx\\ & \overset{\left(c\right)}{=} & m\cdot \int_{R^{n}}\min\left\{q_{0}\cdot p\left(x|\theta_{0}\right),\ldots ,q_{m-1}p\left(x|\theta_{m-1}\right)\right\}\times \\ & & \rho \left(\frac{1}{m}\sum_{i=0}^{m-1}T_{i}\theta_{i}\right)dx\\ & = & m\rho \left(\frac{1}{m}\sum_{i=0}^{m-1}T_{i}\theta_{i}\right)\cdot \int_{R^{n}}\min\left\{q_{0}\cdot p\left(x|\theta_{0}\right),\ldots ,q_{m-1}p\left(x|\theta_{m-1}\right)\right\}dx, \end{array}$$

where in (a), we have used the unitarity (and hence the norm-preserving property) of $\left\{T_{i}\right\}$, as ρ(·) is assumed to depend only on the norm of the error vector; in (b), we used the convexity of ρ, and in (c), we used the fact that $\sum_{i=0}^{m-1}T_{i}=0$, which implies that $\sum_{i=0}^{m-1}T_{i}g_{n}\left[X\right]=0$, thus making the bound independent of the estimator, $g_{n}$. This completes the proof of Theorem 4. □

Theorem 1 is a special case where m=2, $T_{0}=I$, and $T_{1}=-I$, where *I* is the d×d identity matrix. The integral associated with the lower bound of Theorem 4 might not be trivial to evaluate in general for m≥3. However, there are some choices of the auxiliary parameters that may facilitate calculations. One such choice is as follows. For some positive integer k<m, take $\theta_{0}=\theta_{1}=\ldots =\theta_{k-1}\overset{▵}{=}\vartheta_{0}$, for some $\vartheta_{0}\in \Theta$, $q_{0}=q_{1}=\ldots =q_{k-1}\overset{▵}{=}Q/k$, for some Q∈(0,1), $\theta_{k}=\theta_{k+1}=\ldots =\theta_{m-1}\overset{▵}{=}\vartheta_{1}$, for some $\vartheta_{1}\in \Theta$, and finally, $q_{k}=q_{k+1}=\ldots =q_{m-1}=\left(1-Q\right)/\left(m-k\right)$. The integrand then becomes the minimum between two functions only, as in Section 3. Denoting α=k/m, the bound then becomes

(96) $$\begin{array}{ccc} R_{n}\left(\Theta \right) & \geq & m\rho \left(\frac{1}{m}\sum_{i=0}^{k-1}T_{i}\left(\vartheta_{0}-\vartheta_{1}\right)\right)\cdot \int_{R^{n}}\min\left\{\frac{Q}{k}\cdot p\left(x|\vartheta_{0}\right),\frac{1-Q}{m-k}\cdot p\left(x|\vartheta_{1}\right)\right\}dx\\ & = & \rho \left(\frac{1}{m}\sum_{i=0}^{k-1}T_{i}\left(\vartheta_{0}-\vartheta_{1}\right)\right)\cdot \int_{R^{n}}\min\left\{\frac{Q}{\alpha }\cdot p\left(x|\vartheta_{0}\right),\frac{1-Q}{1-\alpha }\cdot p\left(x|\vartheta_{1}\right)\right\}dx\\ & = & \rho \left(\frac{1}{m}\sum_{i=0}^{k-1}T_{i}\left(\vartheta_{0}-\vartheta_{1}\right)\right)\cdot \left(\frac{Q}{\alpha }+\frac{1-Q}{1-\alpha }\right)\cdot P_{e}\left(\frac{(1-\alpha )Q}{\left(1-\alpha )Q+\alpha (1-Q\right)},\vartheta_{0},\vartheta_{1}\right). \end{array}$$

Redefining

(97) $$q=\frac{(1-\alpha )Q}{\left(1-\alpha )Q+\alpha (1-Q\right)},$$

we have

(98) $$\frac{Q}{\alpha }+\frac{1-Q}{1-\alpha }=\frac{1}{1-\alpha -q+2\alpha q},$$

and the following corollary to Theorem 2 is obtained.

**Corollary** **2.** *Let the conditions of Theorem 4 be satisfied. Then,*

(99) $$\begin{array}{ccc} R_{n}\left(\Theta \right) & \geq & \underset{\vartheta_{0},\vartheta_{1},\alpha ,q}{\sup}\rho \left(\frac{1}{m}\sum_{i=0}^{k-1}T_{i}\left(\vartheta_{0}-\vartheta_{1}\right)\right)\cdot \frac{P_{e}\left(q,\vartheta_{0},\vartheta_{1}\right)}{1-\alpha -q+2\alpha q}\\ & = & \underset{\vartheta_{0},\vartheta_{1},q}{\sup}\rho \left(\frac{1}{m}\sum_{i=0}^{k-1}T_{i}\left(\vartheta_{0}-\vartheta_{1}\right)\right)\cdot \frac{P_{e}\left(q,\vartheta_{0},\vartheta_{1}\right)}{\min\{q,1-q\}}. \end{array}$$

Note that if *m* is even, $\alpha =\frac{1}{2}$, and $T_{0}=I$; then, we are actually back to the bound of m=2, and so, the optimal bound for even m>2 cannot be worse than our bound of m=2. We do not have, however, precisely the same argument for an odd *m*, but for a large *m*, it becomes immaterial if *m* is even or odd. In its general form, the bound of Theorem 4 is a heavy optimization problem, as we have the freedom to optimize $\theta_{0},\ldots ,\theta_{m-1}$, $T_{0},\ldots ,T_{m-1}$ (under the constraints that they are all unitary and sum to zero), and $q_{0},\ldots ,q_{m-1}$ (under the constraints that they are all non-negative and sum to unity).

Another relatively convenient choice is to take $\theta_{i}=T_{i}^{-1}\theta_{0}$, i=1,…,m−1 to obtain another corollary to Theorem 4:

**Corollary** **3.** *Let the conditions of Theorem 2 be satisfied. Then,*

(100) $$R_{n}\left(\Theta \right)\geq \underset{\theta_{0},T_{0},\ldots ,T_{m-1},q_{0},\ldots ,q_{m-1}}{\sup}m\rho \left(\theta_{0}\right)\cdot \int_{R^{n}}\min\left\{q_{0}p\left(x|\theta_{0}\right),q_{1}p\left(x|T_{1}^{-1}\theta_{0}\right),\ldots ,q_{m-1}p\left(x|T_{m-1}^{-1}\theta_{0}\right)\right\}dx.$$

**Example** **12.** *To demonstrate a calculation of the extended lower bound for m=3, consider the following model. We are observing a noisy signal,*

(101) $$Z_{i}=\vartheta \phi_{i}+\left(\vartheta +\zeta \right)\psi_{i}+N_{i},\;\;\;\;\;i=1,2,\ldots ,n,$$

*where ϑ is the desired parameter to be estimated, ζ is a nuisance parameter, taking values within an interval [−δ,δ] for some δ>0, $\left\{N_{i}\right\}$ are i.i.d. Gaussian random variables with zero mean and variance $\sigma^{2}$, and $\phi_{i}$ and $\psi_{i}$ are two given orthogonal waveforms with $\sum_{i=1}^{n}\phi_{i}^{2}=\sum_{i=1}^{n}\psi_{i}^{2}=n$. Suppose we are interested in estimating ϑ based on the sufficient statistics $X=\frac{1}{n}\sum_{i=1}^{n}Z_{i}\phi_{i}$ and $Y=\frac{1}{n}\sum_{i=1}^{n}Z_{i}\psi_{i}$, which are jointly Gaussian random variables with the mean vector (ϑ,ϑ+ζ) and the covariance matrix $\frac{\sigma^{2}}{n}\cdot I$, I being the 2×2 identity matrix. We denote realizations of (X,Y) by (x,y). Let us also denote θ=(ϑ,ϑ+ζ). Since we are interested only in estimating ϑ, our loss function will depend only on the estimation error of the first component of θ, which is ϑ. Consider the choice m=3 and let $T_{i}$ be counter-clockwise rotation transformations by 2πi/3, i=0,1,2. For a given Δ∈(0,δ], let us select $\theta_{0}=\left(-\Delta ,0\right)$, $\theta_{1}=T_{1}^{-1}\theta_{0}=\left(\Delta /2,\Delta \sqrt{3}/2\right)$, and $\theta_{2}=T_{2}^{-1}\theta_{0}=\left(\Delta /2,-\Delta \sqrt{3}/2\right)$. Finally, let $q_{0}=q_{1}=q_{2}=\frac{1}{3}$. In order to calculate the integral*

$$I=\int_{R^{2}}\min\left\{\frac{1}{3}p\left(x,y|\theta_{0}\right),\frac{1}{3}p\left(x,y|\theta_{1}\right),\frac{1}{3}p\left(x,y|\theta_{2}\right)\right\}dxdy,$$

*the plane $R^{2}$ can be partitioned into three slices over which the integrals contributed are equal. In each such region, the smallest $p(x,y|\theta_{i})$ is integrated. In other words, every $p(x,y|\theta_{i})$ in its turn is integrated over the region whose Euclidean distance to $\theta_{i}$ is larger than the distances to the other two values of θ. For $\theta_{0}=\left(-\Delta ,0\right)$, this is the region $\left\{\left(x,y\right):\;x\geq 0,\;|y|\leq x\sqrt{3}\right\}$. The factor of $\frac{1}{3}$ cancels with the three identical contributions from $\theta_{0}$, $\theta_{1}$, and $\theta_{2}$ due to the symmetry. Therefore,*

(102) $$\begin{array}{ccc} I & = & \int_{0}^{\infty }dx\int_{-x\sqrt{3}}^{x\sqrt{3}}dy\cdot p\left(x,y|\theta_{0}\right)\\ & = & \int_{0}^{\infty }dx\int_{-x\sqrt{3}}^{x\sqrt{3}}\cdot \frac{n}{2\pi \sigma^{2}}\exp\left\{-\frac{n{\left(x+\Delta \right)}^{2}+ny^{2}}{2\sigma^{2}}\right\}dy\\ & = & \sqrt{\frac{n}{2\pi \sigma^{2}}}\cdot \int_{0}^{\infty }\exp\left\{-\frac{n{\left(x+\Delta \right)}^{2}}{2\sigma^{2}}\right\}\left[1-2Q\left(\frac{x\sqrt{3n}}{\sigma }\right)\right]dx. \end{array}$$

*Our next mathematical manipulations in this example are in the spirit of the passage from Theorem 1 to Corollary 1, that is, selecting the test points increasingly close to each other as functions of n, so that the probability of list error would tend to a positive constant. To this end, we change the integration variable x to $u=x\sqrt{n}/\sigma$ and select $\Delta =s\sigma /\sqrt{n}$ for some s≥0 to be optimized later. Then,*

(103) $$\begin{array}{ccc} I & = & \sqrt{\frac{n}{2\pi \sigma^{2}}}\cdot \int_{0}^{\infty }\exp\left\{-\frac{n{\left(u\sigma /\sqrt{n}+s\sigma /\sqrt{n}\right)}^{2}}{2\sigma^{2}}\right\}\left[1-2Q(u\sqrt{3})\right]d\left(\frac{u\sigma }{\sqrt{n}}\right)\\ & = & \frac{1}{\sqrt{2\pi }}\cdot \int_{0}^{\infty }e^{-{\left(u+s\right)}^{2}/2}\cdot \left[1-2Q\left(u\sqrt{3}\right)\right]du. \end{array}$$

*The MSE bound then becomes*

(104) $$\begin{array}{ccc} R_{n}\left(\theta ,g_{n}\right) & \geq & \underset{s\geq 0}{\sup}\left\{3\cdot {\left(\frac{s\sigma }{\sqrt{n}}\right)}^{2}\cdot \frac{1}{\sqrt{2\pi }}\cdot \int_{0}^{\infty }e^{-{\left(u+s\right)}^{2}/2}\cdot \left[1-2Q\left(u\sqrt{3}\right)\right]du\right\}\\ & = & \frac{\sigma^{2}}{n}\cdot \underset{s\geq 0}{\sup}\left\{\frac{3s^{2}}{\sqrt{2\pi }}\cdot \int_{0}^{\infty }e^{-{\left(u+s\right)}^{2}/2}\cdot \left[1-2Q\left(u\sqrt{3}\right)\right]du\right\}\\ & \approx & \frac{0.2514\sigma^{2}}{n}. \end{array}$$

*This bound is not as tight as the corresponding bound of m=2, which results in $0.3314\sigma^{2}/n$, but it should be kept in mind that here, we have not attempted to optimize the choices of $\theta_{0}$, $\theta_{1}$, $\theta_{2}$, $T_{0}$, $T_{1}$, $T_{2}$, $q_{0}$, and $q_{1}$. Instead, we have chosen these parameter values from considerations of computational convenience, just to demonstrate the calculation. This concludes Example 12.*

Finally, a comment is in order regarding the possible extensions of Section 4 to the vector case. Such extensions are conceptually straightforward whenever the loss function of the error vector is given by the sum of losses associated with the different components of the error vector. Most notably, when the loss function is the MSE, $\rho \left(\epsilon \right)={∥\epsilon ∥}^{2}$, each component of the estimation error can be handled separately by the methods of Section 4. Of course, the two or three test points should be chosen such that they differ in all components of the parameter vector. In the case of three test points, it makes sense to select them equally spaced along a certain straight line of a general direction in Θ. We will not pursue any further this extension in the framework of this work.

## Data Availability

The original contributions presented in the study are included in the article, further inquiries can be directed to the corresponding author.

## Appendix A

**Proof** **of Equation (44).** Consider the Taylor series expansion,

(A1) $$s\left(t,\theta +\delta \right)=s\left(t,\theta \right)+\delta \cdot \dot{s}\left(t,\theta \right)+\frac{\delta^{2}}{2}\cdot \ddot{s}\left(t,\theta \right)+o\left(\delta^{2}\right),$$

where $\dot{s}\left(t,\theta \right)$ and $\ddot{s}\left(t,\theta \right)$ are the first two partial derivatives of s(t,θ) w.r.t. θ. Correlating both sides with s(t,θ) yields

(A2) $$\int_{0}^{T}s\left(t,\theta \right)s\left(t,\theta +\delta \right)dt=E+\delta \cdot \int_{0}^{T}s\left(t,\theta \right)\dot{s}\left(t,\theta \right)dt+\frac{\delta^{2}}{2}\cdot \int_{0}^{T}s\left(t,\theta \right)\ddot{s}\left(t,\theta \right)dt+o\left(\delta^{2}\right).$$

Now,

(A3) $$\int_{0}^{T}s\left(t,\theta \right)\dot{s}\left(t,\theta \right)dt=\frac{1}{2}\cdot \frac{\partial }{\partial \theta }\left\{\int_{0}^{T}s^{2}\left(t,\theta \right)dt\right\}=\frac{1}{2}\cdot \frac{\partial E}{\partial \theta }=0,$$

since the energy *E* is assumed independent of θ. Also, since $\frac{\partial^{2}E}{\partial \theta^{2}}=0$, we have

(A4) $$\begin{array}{ccc} 0 & = & \frac{\partial^{2}}{\partial \theta^{2}}\left\{\int_{0}^{T}s^{2}\left(t,\theta \right)dt\right\}\\ & = & \frac{\partial }{\partial \theta }\left\{2\cdot \int_{0}^{T}s\left(t,\theta \right)\dot{s}\left(t,\theta \right)dt\right\}\\ & = & 2\cdot \int_{0}^{T}{\dot{s}}^{2}\left(t,\theta \right)dt+2\cdot \int_{0}^{T}s\left(t,\theta \right)\ddot{s}\left(t,\theta \right)dt, \end{array}$$

which yields

(A5) $$\int_{0}^{T}s\left(t,\theta \right)\ddot{s}\left(t,\theta \right)dt=-\int_{0}^{T}{\dot{s}}^{2}\left(t,\theta \right)dt,$$

and so,

(A6) $$\int_{0}^{T}s\left(t,\theta \right)s\left(t,\theta +\delta \right)dt=E-\frac{\delta^{2}}{2}\int_{0}^{T}{\dot{s}}^{2}\left(t,\theta \right)dt+o\left(\delta^{2}\right),$$

and hence,

(A7) $$\varrho \left(\theta ,\theta +\Delta \right)=1-\frac{\delta^{2}}{2E}\int_{0}^{T}{\dot{s}}^{2}\left(t,\theta \right)dt+o\left(\delta^{2}\right).$$

□

## Appendix B

**Proof** **of Lemma 1.** First, observe that

(A8) $$\sum_{i=1}^{k}a_{i}=\sum_{i=1}^{k}r_{i}\cdot \frac{a_{i}}{r_{i}}\leq \max\left\{\frac{a_{1}}{r_{1}},\ldots ,\frac{a_{k}}{r_{k}}\right\},$$

and since the inequality $\sum_{i=1}^{k}a_{i}\leq \max\left\{a_{1}/r_{1},\ldots ,a_{k}/r_{k}\right\}$ applies to all r∈S, it also applies to the infimum of $\max\{a_{1}/r_{1},\ldots ,a_{k}/r_{k}\}$ over S, thus establishing the inequality “≤” between the two sides. To establish the “≥” inequality, $r^{*}\in S$ is defined to be the vector whose components are given by $r_{i}^{*}=a_{i}/\sum_{j=1}^{k}a_{j}$. Then,

(A9) $$\underset{(r_{1},\ldots ,r_{k})\in S}{\inf}\max\left\{\frac{a_{1}}{r_{1}},\ldots ,\frac{a_{k}}{r_{k}}\right\}\leq \max\left\{\frac{a_{1}}{r_{1}^{*}},\ldots ,\frac{a_{k}}{r_{k}^{*}}\right\}=\sum_{i=1}^{k}a_{i}.$$

This completes the proof of Lemma 1. □
